# Myopathology and Immune Profile of Granulomatous Myositis in Sarcoid Myopathy

**DOI:** 10.1111/nan.70040

**Published:** 2025-09-10

**Authors:** Nikolas Ruffer, Iago Pinal‐Fernandez, Felix Kleefeld, Marie‐Therese Holzer, Hans‐Hilmar Goebel, Anne Schänzer, Maria Casal‐Dominguez, Ina Kötter, Norman Görl, Florian Masuhr, Rieke Alten, Eckart Braasch, Albert Grüger, Jörg Müller, Thomas Lempert, Andreas Krause, Tobias B. Huber, Teerin Liewluck, Andrew L. Mammen, Werner Stenzel, Corinna Preusse, Udo Schneider, Martin Krusche

**Affiliations:** ^1^ Division of Rheumatology and Systemic Inflammatory Diseases, III. Department of Medicine University Medical Center Hamburg‐Eppendorf Hamburg Germany; ^2^ Department of Neuropathology Charité – Universitätsmedizin Berlin, corporate member of Freie Universität Berlin and Humboldt‐Universität zu Berlin Berlin Germany; ^3^ Muscle Disease Section, National Institute of Arthritis and Musculoskeletal and Skin Diseases National Institutes of Health Bethesda Maryland USA; ^4^ Department of Neurology Johns Hopkins University School of Medicine Baltimore Maryland USA; ^5^ Department of Neurology Charité – Universitätsmedizin Berlin, corporate member of Freie Universität Berlin and Humboldt‐Universität zu Berlin Berlin Germany; ^6^ Department of Neurology, Heimer Institute for Muscle Research University Hospital Bergmannsheil, Ruhr‐University Bochum Bochum Germany; ^7^ Institute of Neuropathology Justus‐Liebig‐University Gießen Gießen Germany; ^8^ Rheumazentrum Nordwest Wismar Germany; ^9^ Department of Neurology Bundeswehrkrankenhaus Berlin Berlin Germany; ^10^ Department of Internal Medicine and Rheumatology Schlosspark‐Klinik Berlin Germany; ^11^ Department of Nephrology, Rheumatology and Endocrinology Werner Forßmann Klinikum Eberswalde Eberswalde Germany; ^12^ Department of Neurology Martin Gropius Krankenhaus Eberswalde Germany; ^13^ Department of Neurology Vivantes Klinikum Spandau Berlin Germany; ^14^ Department of Neurology Schlosspark‐Klinik Berlin Germany; ^15^ Department of Rheumatology, Clinical Immunology and Osteology Immanuel Hospital Berlin Berlin Germany; ^16^ III. Department of Medicine University Medical Center Hamburg‐Eppendorf Hamburg Germany; ^17^ Division of Neuromuscular Medicine, Department of Neurology Mayo Clinic Rochester Minnesota USA; ^18^ Department of Medicine Johns Hopkins University School of Medicine Baltimore Maryland USA; ^19^ Department of Pediatric Neurology Charité – Universitätsmedizin Berlin, corporate member of Freie Universität Berlin and Humboldt‐Universität zu Berlin Berlin Germany; ^20^ Department of Rheumatology and Clinical Immunology Charité – Universitätsmedizin Berlin, corporate member of Freie Universität Berlin and Humboldt‐Universität zu Berlin Berlin Germany

**Keywords:** giant cells, granuloma, inclusion body myositis, muscle biopsy, myositis

## Abstract

**Aims:**

Sarcoid myopathy (SaM) is characterised by granulomatous myositis (GM) and can overlap with inclusion body myositis (IBM), a late‐onset chronic idiopathic inflammatory myopathy with a still enigmatic pathogenesis. As GM can occur in different clinical contexts, we aimed to examine the histomorphologic features and gene expression profiles in cases of definite SaM that may inform diagnostic and therapeutic considerations.

**Methods:**

We performed a multidimensional characterisation of muscle biopsy specimens from patients with ‘pure SaM’ (n=17), SaM with concomitant IBM (SaM‐IBM) (n=2), including histopathologic and ultrastructural analysis in addition to quantitative real‐time polymerase chain reaction. Additionally, bulk RNA sequencing was performed on 38 muscle biopsy specimens from patients with SaM (including SaM‐IBM) (n=30) and NGI (n=8).

**Results:**

Histopathological analysis revealed a pattern of endomysial and perimysial granulomatous inflammation frequently extending to the fascia, endomysial fibrosis, muscle fibre atrophy, variation in muscle fibre size and capillary thickening. Findings from immunohistochemical studies established Chitinase 1 as a pure giant cell marker in SaM. On a subcellular level, ‘pure SaM’ was characterised by focal accumulation of large swollen mitochondria with rare cristae but an absence of irregular cristae. Transcriptomic analysis of patients with SaM confirmed markedly elevated expression of both type 1 and type 2 human leukocyte antigen molecules. Macrophage activity markers were highly elevated. Consistent with histologic findings, CHIT1 was specifically overexpressed in SaM samples but not in ‘pure IBM’ muscle biopsy specimens.

**Conclusions:**

SaM is characterised by a stereotypical appearance at the histopathologic level and disease‐specific immune dysregulation that involves macrophage function and maturation. SaM‐IBM represents a noteworthy overlap syndrome that shares multiple dysregulated immune pathways with ‘pure SaM’.

## Summary

- SaM is characterised by endomysial and perimysial granulomatous inflammation frequently extending to the fascia, endomysial fibrosis, muscle fibre atrophy, variation in muscle fibre size and capillary thickening.
- Chitinase 1 represents a pure giant cell marker in SaM.
- SaM is characterised by a disease‐specific immune dysregulation that involves macrophage function and maturation.

## Graphical abstract

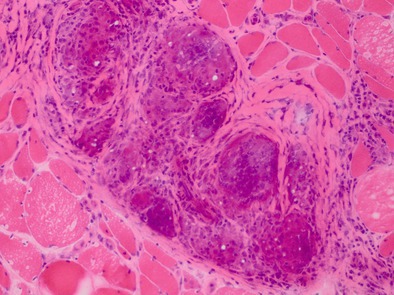

## 1 Introduction

Clinically overt muscle involvement is a rare manifestation of sarcoidosis, a multiorgan granulomatous disease that predominantly affects the lung and lymph nodes [1, 2]. Common symptoms of sarcoid myopathy (SaM) include muscle weakness and myalgia, whereas serum creatine kinase (CK) levels are normal in most cases [2, 3]. The clinical course of SaM is heterogeneous and frequently includes relapses [1]. Specifically, four clinical patterns have been described in SaM: nodular, smouldering, myopathic and combined neurogenic/myopathic phenotypes [1]. However, SaM may be asymptomatic [4].

Interestingly, SaM may overlap with antisynthetase syndrome and inclusion body myositis (IBM) [5, 6, 7, 8]. The overlap syndrome SaM‐IBM is associated with severe motor deficits and poor treatment response, comparable to sporadic IBM [8, 9]. Interestingly, accelerated progression to IBM has been recently discussed in granulomatous myositis (GM) associated with sarcoidosis (compared to isolated GM) [8], which raises the question of specific inflammatory disease drivers in SaM. Notably, the presence of IBM in GM associated with sarcoidosis seems to be higher than in the general population [2].

Muscle inflammation in SaM is characterised by non‐necrotising granuloma formation that involves a distinct polarised type of macrophage implicated in fibrosis and repair mechanisms [10, 11]. Additionally, predominant infiltration of CD4^+^ lymphocytes, which is pronounced in the vicinity of the granulomas, has been reported [12, 13, 14, 15]. Immunohistochemical studies also revealed sarcolemmal upregulation of major histocompatibility complex (MHC) Class I and MHC Class II [10, 12, 15, 16]. Endomysial fibrosis, signs of atrophy and variation in muscle fibre size suggest a chronic and potentially subclinical process of myoinflammation [12, 13, 14, 15, 17]. Of note, studies investigating the pathological and immunological features of SaM are scarce and based on small cohorts [2]. Whether specific morphologic findings differentiate SaM from other forms of GM [18] that may occur in different clinical contexts remains an important question.

The immune pathogenesis of sarcoidosis is thought to involve a dysregulated immune response against a currently unidentified antigen, leading to inflammation, granuloma formation and subsequent organ fibrosis [19]. Recently, the elevated expression of transposon‐like human element 1B (*THE1B*) transcripts has been implicated in the pathogenesis of SaM [20].

Our previous work demonstrated that granuloma formation in SaM depends on Th2‐M2 polarisation and significant expression of DAP12/TYROB (DNAX‐activation protein 12/tyrosine protein tyrosine kinase binding protein) and transforming growth factor β (TGF‐β) in macrophages [11]. Subsequent findings from spatial transcriptomic studies suggest that granulomas act as paracrine structures, which mediate proinflammatory and profibrotic signalling, including tumour necrosis factor α (TNFα) and TGF‐β pathway activation [21]. The latter has been linked to skeletal muscle fibrosis in murine models of muscular dystrophy [22]. Granulomatous lesions in SaM also demonstrate upregulated expression of interferon‐γ (IFNG), interleukin (IL)12 and IL18 [23]. Furthermore, muscle fibre destruction may result from direct invasion of inflammatory cells and has been linked to expression of proteases (cathepsin B, calpain II and ubiquitin‐proteasome) in macrophages and epithelioid cells within granulomas [14].

In cardiac sarcoidosis, GPNMB^+^ (transmembrane glycoprotein NMB) multinucleated giant cells encased by macrophages expressing MHC Class II showed subsequent mammalian target of rapamycin activation and have been identified as a distinct morphological feature [24]. Interestingly, these findings have not been studied in the skeletal muscle of patients with SaM.

As GM can occur in different clinical contexts, we studied the histomorphologic features and gene expression profiles in cases of definite SaM that may inform diagnostic and therapeutic considerations. Specifically, previously established biomarkers of extramuscular sarcoidosis were analysed in the skeletal muscle tissue of patients with SaM. Also, the noteworthy overlap of SaM‐IBM and well‐known mitochondrial alterations in IBM spectrum disease raise the question of shared immune pathologies [25, 26].

We performed multidimensional morphological and immune phenotyping of the skeletal muscle tissue and compared immunological trajectories in ‘pure SaM’, ‘SaM‐IBM’ and ‘pure IBM’ to unveil the myopathologic features and pathophysiology of muscle inflammation in this enigmatic disease. Comparison to IBM is of particular interest as IBM, on one hand, has been intensely studied on a clinical, morphological and immunological level, but on the other hand, it is not well understood in terms of therapy refractoriness.

## 2 Methods

### 2.1 Patients and Muscle Biopsy Specimens

The pathology reports of skeletal muscle biopsies from the Department of Neuropathology at Charité – Universitätsmedizin Berlin (Germany) between 2008 and 2024 were retrospectively reviewed. Reports explicitly stating morphologic findings of granulomatous inflammation (*n* = 25) were selected for subsequent histopathologic evaluation and review of the patients' charts. Muscle biopsy specimens from patients with (a) histopathological signs of GM, (b) at least one extramuscular organ manifestation compatible with sarcoidosis (e.g., bilateral hilar lymphadenopathy) and (c) a clinical diagnosis of sarcoidosis based on patient charts were included in this study. Clinical data at the time of the muscle biopsy were retrieved and evaluated by one rheumatologist (N.R.) and one neurologist (F.K.).

Six muscle biopsies from the initial Berlin cohort did not meet the inclusion criteria because signs of GM were absent (1 case of granulomatous vasculitis) or patient chart review suggested an alternative diagnosis (4 cases of non‐specific GM and 1 case of myasthenia gravis‐associated GM). In total, the muscle biopsy specimens from 19 patients with SaM (17 ‘pure SaM’ cases and 2 SaM‐IBM cases) were included in this study for morphologic analysis.

Muscle biopsy specimens of 18 patients with SaM (from the Berlin cohort) were analysed via quantitative real‐time polymerase chain reaction (qRT‐PCR). For comparative analysis, muscle biopsies from patients with non‐specific granulomatous inflammation (NGI; *n* = 6), IBM patients (*n* = 12) and non‐disease controls (NDC; *n* = 5) were used. NDC biopsies were obtained from patients who underwent biopsy for diagnostic purposes, and routine diagnostic studies failed to establish significant alterations of the skeletal muscle, including signs of inflammation or any associated laboratory or clinical findings (e.g., CK elevation and autoantibodies).

For bulk RNA sequencing purposes, muscle biopsy specimens from patients with GM were collected from Institutional Review Board (IRB)–approved longitudinal cohorts at the National Institutes of Health (Bethesda, MD), Johns Hopkins Myositis Center (Baltimore, MD), Vall d'Hebron Hospital (Barcelona), Clinic Hospital (Barcelona), Mayo Clinic (Rochester, MN), Justus‐Liebig‐University (Gießen) and Charité – Universitätsmedizin (Berlin) (Figure S1) [27]. In total, bulk RNA sequencing was performed on 38 muscle biopsy specimens from patients with SaM (including SaM‐IBM) and NGI at the National Institutes of Health (NIH) (Bethesda, MD, USA). Additional muscle biopsy specimens from patients with different types of myositis and genetic myopathies were used for comparative analyses as previously described [28, 29, 30, 31, 32]. The present work focused on the subset of muscle biopsy specimens from patients with SaM. The transcriptomic profiles of GM have been studied in another project [27].

The Charité ethics committee had granted ethical approval (EA2/163/17, AZ 07/09) for this study. A Material Transfer Agreement (MTA) was established between the collaborators.

### 2.2 Tissue Preparation

18/19 SaM (including SaM‐IBM) muscle biopsy specimens and 12/12 IBM muscle biopsy specimens were cryopreserved immediately after removal at −80°C prior to morphologic analysis. Cryostat sections (8 μm thick) were stained according to local standardised procedures [33] and international recommendations [34, 35]. One of the SaM muscle biopsy specimens had been fixed and embedded in paraffin, which limited staining procedures and, therefore, evaluation of the tissue.

Diagnostic conventional histology and enzyme histochemistry reactions (haematoxylin and eosin [H&E], Gömöri trichrome [Gö], Elastic van Gieson [EvG], acid phosphatase [AcP] non‐specific esterase [NSE], cytochrome c oxidase/succinate dehydrogenase [COX/SDH], SDH, ATPases pH [4.3, 4.6 and 9.4]) were performed. Immunohistochemical staining procedures were carried out according to standardised protocols. The following primary antibodies were used: MHC Class I, MHC Class II, C5b‐9, CD4, CD8, CD20, CD31, CD45, CD68, CD138, CD163, chitinase 1 (CHIT1), GPNMB, DAP12/TYROB and ubiquitin‐binding protein p62 (p62). Table S2 details the primary antibodies, dilution, species, company and purchase number used.

Appropriate positive and negative controls were used where necessary, and a physiological control or a normal muscle was used as a negative control for all reactions [33].

### 2.3 Morphologic Analysis

Based on conventional and immunohistochemical stains, we performed a qualitative analysis of the following findings (present/absent): localisation of granulomatous inflammation (endomysial, perimysial and fascia), muscle fibre necrosis, muscle fibre atrophy, variations of muscle fibre size, presence of targetoid fibres, presence of COX‐negative fibres, presence of checkerboard pattern, presence of internalised myonuclei, presence of myophagocytosis, complement depositions of C5b‐9 (muscle fibres and capillaries), capillary thickening, p62 positivity and presence of focal CD20^+^ lymphocytic infiltrates.

In addition, a semiquantitative analysis of additional aspects was performed (+ mildly positive/present; ++ moderately positive/present; +++ strongly positive/present; − negative/not present): endomysial fibrosis, upregulation of MHC Class I and MHC Class II (granuloma and muscle fibres), inflammatory infiltration by lymphomononuclear cells (CD20, CD68, CD138 and CD163), GPNMB (granuloma and interstitium), DAP12/TYROB (granuloma and interstitium), CHIT1 (giant cells).

Quantitative assessment of inflammatory infiltrates with counting of 10 high power fields (HPFs) (based on the microscope used and the respective oculars (Olympus WH10x‐H/22) ≙ 0.16 mm^2^) was performed for CD4, CD8 and CD45.

### 2.4 RNA Extraction and Quantitative Real‐Time Polymerase Chain Reaction (qRT‐PCR)

Analysis of gene expression was performed by quantitative real‐time polymerase chain reaction (qRT‐PCR) as previously reported [33, 36]. Briefly, TRIzol/chloroform was used for total RNA extraction, followed by reverse RNA transcription and generation of cDNA using the High‐Capacity cDNA Archive Kit (Applied Biosystems, Foster City, California, USA) according to the manufacturer's protocol. An Applied Biosystems QuantStudio 6 Flex Real‐Time PCR System (Thermo Fisher, Waltham, Massachusetts, USA; running conditions: 95°C 0:20, 95°C 0:01, 60°C 0:20, 40 cycles) was used for qRT‐PCR reactions. Target genes were normalised to the expression of the housekeeping gene *PGK1* Hs99999906_m1. Genes analysed were *CCL5* Hs00982282_m1, *CXCL10* Hs00171042_m1, *DAP12/TYROB* Hs00182426_m1, *GPNMB* Hs01095669_m1, *HLA‐DRA* Hs00219575_m1, *HLA‐DRB1* Hs02339733_m1, *IFNG* Hs00989291_m1, *IL1B* Hs01555410_m1, *IL12B* Hs01011518_m1, *IL6* Hs00985639_m1, *CAMLG* Hs00266143_m1, *PERK/EIF2AK3* Hs00984003_m1, *SIGLEC1/CD169* Hs00224991_m1, *STAT1* Hs01013989_m1, *TGFB* Hs00998133_m1 and *TNFA* Hs00174128_m1.

Mitochondrial DNA (mtDNA) copy number was determined by quantifying gene expression of the mitochondrial‐encoded gene *MTND1* relative to the nuclear‐encoded *B2M* gene using the ΔCt method (ΔCt = Ct_*B2M*_ − Ct_*MTND1*_). Accounting for the fact that the nuclear genome is diploid (two copies per cell), we further calculated the relative mtDNA copy number: mtDNA copy number = 2 * 2^(ΔCt).

Gene expression is illustrated by the logarithmic fold‐change (RQ = 2^−ddCt^) values between patients and NDC. For gene transcripts that were not expressed in NDC, dCt values are shown.

### 2.5 Transmission Electron Microscopy (TEM)

Ultrastructural evaluation of the three entities (5 cases of SaM, 2 cases of SaM‐IBM and 25 cases of IBM) using transmission electron microscopy (TEM) was performed. Muscle biopsy specimens were fixed and embedded according to standard protocols as previously described [36]. Briefly, 70‐nm ultrathin sections were cut using an ultramicrotome and an Ultra 35° diamond knife (Diatome), stretched with xylene vapour, collected onto pioloform‐coated slot grids and then stained with lead citrate. Standard TEM was performed using a Zeiss 901 microscope in conjunction with a 2‐k CCD camera (TRS).

### 2.6 RNA Sequencing

Bulk RNA sequencing was performed on frozen muscle biopsy specimens, as previously described [28, 29, 30, 31, 32]. Briefly, muscle biopsies were immediately flash‐frozen and stored at −80°C at all contributing centres. The samples were then transported on dry ice to the NIH, where they were uniformly processed for library preparation and analysis. RNA was extracted using TRIzol reagent. Libraries were prepared using either the NeoPrep system following the TruSeq Stranded mRNA Library Prep protocol (Illumina, San Diego, CA) or the NEBNext Poly(A) mRNA Magnetic Isolation Module and Ultra II Directional RNA Library Prep Kit for Illumina (New England BioLabs, ref. #E7490 and #E7760).

### 2.7 Statistical Analysis

For qRT‐PCR analysis, sample sizes were not based on a priori power calculation but based on previous studies [36]. Statistical tests are only used for description, not statistical testing purposes. Data are presented as violin plots. Quantitative variables of mRNA transcripts were analysed by the Mann–Whitney *U* test or Kruskal–Wallis test, followed by Bonferroni–Dunn correction for multiple comparisons. The level of significance was set at *p* < 0.05. GraphPad Prism 9.02 software (GraphPad Software Inc., La Jolla, CA, USA) was used for statistical analysis and visualisation.

For RNA sequencing analysis, sequencing reads were processed for demultiplexing using bcl2fastq/2.20.0 and pre‐processed with fastp/0.23.4 for quality control. Gene abundance was quantified with Salmon/1.5.2. For visualisation purposes, count data were normalised using the Trimmed Mean of M‐values (TMM) method implemented in edgeR/4.2.1. Differential gene expression analysis was conducted with limma/3.60.6, and multiple comparisons were adjusted using the Benjamini–Hochberg method where applicable. Graphical outputs were generated using R and Python software.

## 3 Results

### 3.1 Clinical Features of SaM

The Berlin cohort included patients with SaM, identified retrospectively at the Department of Neuropathology at Charité – Universitätsmedizin Berlin (Germany). In total, 19 muscle biopsies from patients with SaM, including two cases of SaM‐IBM (extramuscular features: Case 1 had lung involvement and lymphadenopathy, and Case 2 had peripheral neuropathy), were analysed. The most frequent biopsy sites were the quadriceps femoris (12/19; 63.1%) and gastrocnemius muscle (3/19; 15.7%). The presence of adjacent fascial tissue was noted in 6/19 (31.5%) of the biopsy specimens. 3/19 (15.7%) of the patients underwent a combined muscle and nerve biopsy.

The median age at the time of muscle biopsy was 59 years (range: 44–75), and the majority of the patients were female (13/19; 68.4%). The median number of extramuscular manifestations included mainly two (maximum three) organs, with lung mostly affected (10/19; 52.6%), followed by the lymphatic (5/19; 26.3%) and peripheral nervous system (5/19; 26.3%). Two of the patients were diagnosed with concomitant IBM based on histopathology (Figure 2), ultrastructural features (Figure 4) and clinical phenotype. More than half of the patients (10/18; 55.5%) had significant muscle weakness at the time of the muscle biopsy. Of note, CK activity was normal in about half of the cases (9/17; 52.9%), whereas an elevated soluble IL‐2 receptor concentration (sIL‐2R) was noted in 9/13 (69.2%) patients and increased levels of angiotensin‐converting enzyme activity were present in a third of the patients (3/9; 33.3%).

Table 1 summarises the clinical features of the enrolled patients from the Berlin cohort.

**TABLE 1:** Clinical and biopsy features of patients with ‘pure sarcoid myopathy’ (SaM) and overlapping inclusion body myositis (SaM‐IBM) in the studied cohort (Berlin cohort).

Median age years (range)	59 (44–75)
Sex ratio (male/female)	6/13
Features of muscle biopsy specimens	
Biopsy site	
Quadriceps femoris muscle	12 (63.1%)
Gastrocnemius muscle	3 (15.7%)
Biceps muscle	1 (5.2%)
Temporalis muscle	1 (5.2%)
Not specified	2 (10.4%)
Presence of fascial tissue	6/19 (31.5%)
Combined muscle/nerve biopsy	3/19 (15.7%)
Organ involvement	
Lung	10/19 (52.6%)
Lymphadenopathy	5/19 (26.3%)
Peripheral neuropathy	5/19 (26.3%)
Cardiac involvement	3/19 (15.7%)
Skin	2/19 (10.5%)
Eye	2/19 (10.5%)
Joint	2/19 (10.5%)
Central nervous system	1/19 (5.2%)
Renal involvement	1/19 (5.2%)
Concomitant inclusion body myositis phenotype	2/19 (10.5%)
Median number of extramuscular manifestations	2
Clinical signs and symptoms	
Muscle weakness	10/18 (55.5%)
Muscle atrophy	4/18 (22.2%)
Myalgia	4/18 (22.2%)
Laboratory investigations	
Increased creatine kinase activity	8/17 (47.0%)
Elevated soluble interleukin‐2 receptor concentration	9/13 (69.2%)
Increased angiotensin converting enzyme activity	3/9 (33.3%)
Management	
Glucocorticoids	17/17 (100%)
Methotrexate	6/17 (35.2%)
Azathioprine	4/17 (23.5%)
Infliximab	1/17 (5.8%)
Cyclophosphamide	1/17 (5.8%)
Intravenous immunoglobulin	1/17 (5.8%)

*Note:* Percentages refer to reported information based on patient charts (*n*/reported cases) (%).

### 3.2 Histopathologic Features of SaM and IBM Overlap (SaM‐IBM)

**FIGURE 1:**
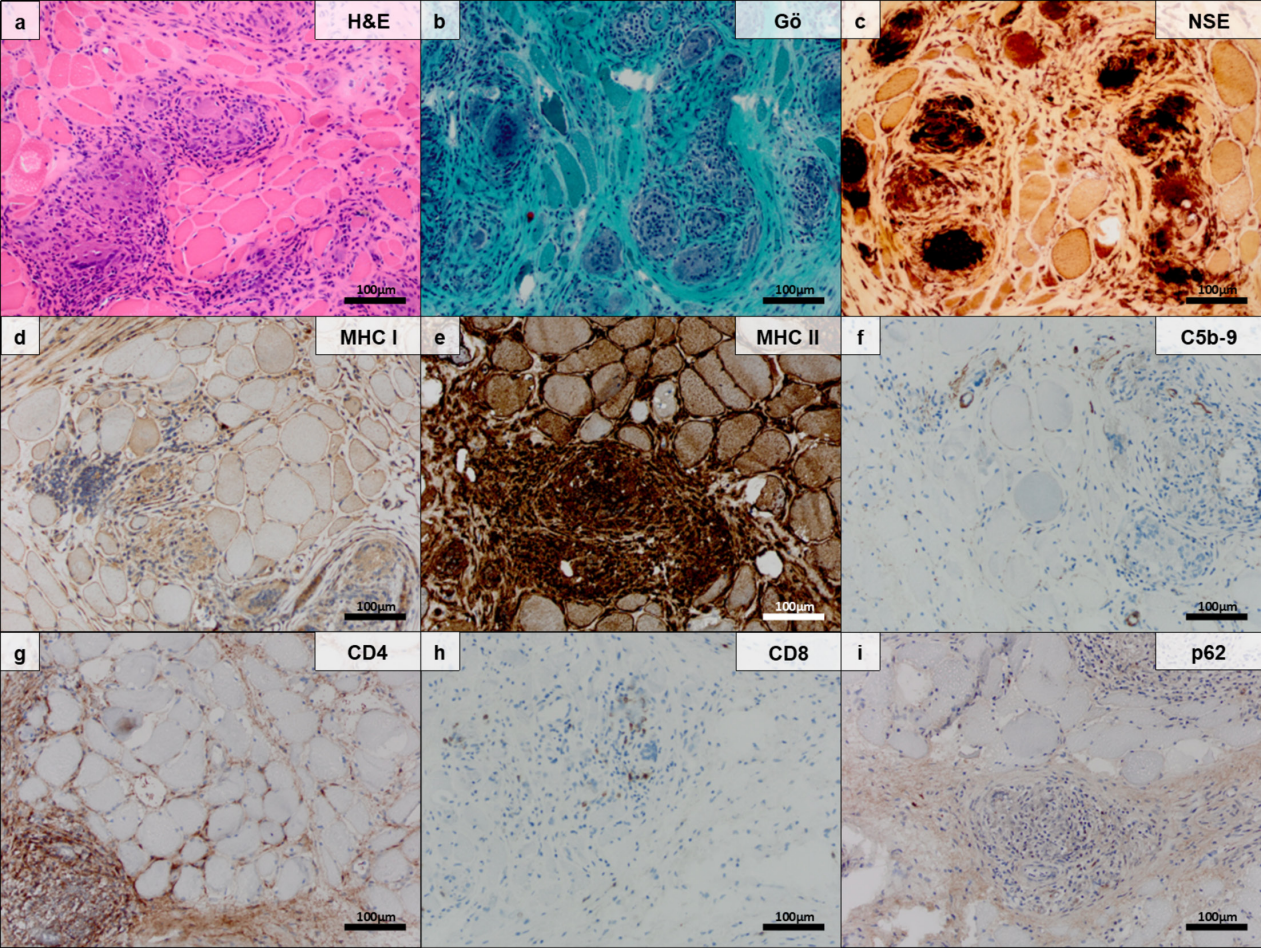
Myopathologic features of a representative ‘pure sarcoid myopathy’ (SaM) muscle biopsy specimen (original magnification 200×; scale bar 100 μm). Histopathologic evaluation revealed granulomatous myositis and concomitant myopathic features including endomysial fibrosis, muscle fibre atrophy, variations of muscle fibre size and capillary thickening. Endomysial granuloma containing some giant cells as well as histiocytes/macrophages and T cells infiltrating the parenchymal arrangement of atrophic myofibres (haematoxylin and eosin staining) (a). Endomysial granuloma and some isolated giant cells, as well as myofibre necrosis, are present in severely fibrotic skeletal muscle tissues, including remnants of small atrophic myofibre, some flattened and triangular (Gömöri trichrome and non‐specific esterase preparations) (b,c). Macrophages/histiocytes and giant cells, as well as the sarcolemma of myofibres, are strongly positive for major histocompatibility complex (MHC) Class I (d) and MHC Class II (e) (MHC Class I and MHC Class II immunohistochemistry). No major complement deposition on myofibres or capillaries is seen in the majority of cases (C5b‐9/MAC immunohistochemistry) (f). The majority of T cells in the vicinity of granulomas are CD4^+^ and less frequently CD8^+^ as well (CD4 and CD8 immunohistochemistry) (g,h). Autophagic material in myofibres, as detected by ubiquitin‐binding protein p62 (p62), is absent in SaM (p62 immunohistochemistry) (i).

Morphologic analysis of SaM muscle biopsy specimens from the Berlin cohort identified a stereotypical pattern (Figure 1) that was characterised by granulomatous inflammation of the endomysium (19/19; 100%), endomysial fibrosis (17/19; 89.4%), muscle fibre atrophy (12/19; 63.1%) with variations of muscle fibre size (17/19; 89.4%) and vessel wall thickening of capillaries in the endomysium (17/18; 94.4%). Endomysial fibrosis was pronounced in most cases (11/19; 57.8%), indicating a chronic process. Additional extension of the granulomatous inflammation to the perimysium (8/19; 42.1%) and fascial involvement (4/6; 66.6%) was frequently observed. In addition, myofibre necrosis (6/19; 31.5%), internal myonuclei (6/19; 31.5%) and myophagocytosis (5/19; 26.3%), often tunnel‐like (in the centre of the sarcoplasm), were present in a significant proportion of cases (Table 2).

**TABLE 2:** Qualitative assessment of myopathologic features of ‘pure sarcoid myopathy’ (SaM) and overlapping inclusion body myositis (SaM‐IBM) in the studied cohort (Berlin cohort).

Granulomatous inflammation	
Endomysial	19/19 (100%)
Perimysial	8/19 (42.1%)
Fascia	4/6 (66.6%)
Endomysial fibrosis	17/19 (89.4%)
Muscle fibre necrosis	6/19 (31.5%)
Muscle fibre atrophy	12/19 (63.1%)
Variations of muscle fibre size	17/19 (89.4%)
Targetoid fibres	7/19 (36.8%)
Age‐inappropriate COX‐negative (SDH‐positive) fibres	3/19 (15.7%)
Checkerboard pattern of muscle fibres	16/18 (88.8%)
Internalised myonuclei	6/19 (31.5%)
Myophagocytosis	5/19 (26.3%)
Complement deposition of C5b‐9	
Muscle fibres	5/18 (27.7%)
Capillaries	1/18 (5.5%)
Capillary vessel wall thickening	17/18 (94.4%)
p62 positive aggregate within muscle fibres	2/16 (12.5%)
Focal CD20^+^ lymphocytic infiltrates	4/18 (22.2%)
Focal CD138^+^ lymphocytic infiltrates	2/18 (11.1%)

Sarcolemmal staining of myofibres was found in all biopsies for MHC Class I (18/18; 100%) and in the vast majority of biopsies for MHC Class II (17/19; 89.4%) as well (Table 3). Inflammatory cells within the granulomas also stained positive for MHC Class I (18/18; 100%) and MHC Class II (19/19; 100%) in all cases. Staining with GPNMB and DAP12/TYROB specifically highlighted granulomas including giant cells and macrophages/histiocytes as well as epithelioid cells in all cases (16/16; 100%), whereas macrophages in the vicinity that were not part of the granulomas were less prominently stained (Figure 3). CHIT1 was positive on giant cells within granulomas, whereas monocytes were not stained (Figure 3). Double immunofluorescence demonstrated co‐staining of giant cells by fusion‐competence markers DAP12/TYROB and CHIT1 (Figure S4). Co‐staining of CD68 and CHIT1 in giant cells subsequently identified CHIT1 as a pure giant cell marker in granulomas of SaM (Figure S5).

**TABLE 3:** Semiquantitative and quantitative analysis of myopathologic features in ‘pure sarcoid myopathy’ (SaM) and overlapping inclusion body myositis (SaM‐IBM) in the studied cohort (Berlin cohort).

Semiquantitative analysis	
Endomysial fibrosis	− 2/19; + 6/19; ++ 4/19; +++ 7/19
CD68	− 0/19; + 0/19; ++ 1/19; +++ 18/19
CD163	− 0/15; + 1/15; ++ 1/15; +++ 13/15
MHC Class I	
Granuloma	− 0/17; + 1/17; ++ 2/17; +++ 14/17
Muscle fibres	− 0/18; + 1/18; ++ 4/18; +++ 13/18
MHC Class II	
Granuloma	− 0/19; + 0/19; ++ 3/19; +++ 16/19
Muscle fibres	− 2/19; + 6/19; ++ 3/19; +++ 8/19
GPNMB granuloma	− 0/16; + 0/16; ++ 1/16; +++ 15/16
DAP12/TYROB granuloma	− 0/16; + 0/16; ++ 4/16; +++ 12/16
CHIT1 giant cells	− 0/16; + 0/16; ++ 0/16; +++ 16/16

Quantitative analysis of inflammatory infiltrates	
Mean (SD) CD4^+^ lymphocytes count per HPF	24 (12.37)
Mean (SD) CD8^+^ lymphocytes count per HPF	22 (12.96)
Mean CD4^+^/CD8^+^ ratio	1.39 (*p* > 0.05)
Mean (SD) CD45^+^ lymphocytes count per HPF	42 (21.5)

*Note:* Semiquantitative analysis was performed as follows: + mildly positive/present; ++ moderately positive/present; +++ strongly positive/present; − negative/not present.

Abbreviation: SD, standard deviation.

**FIGURE 2:**
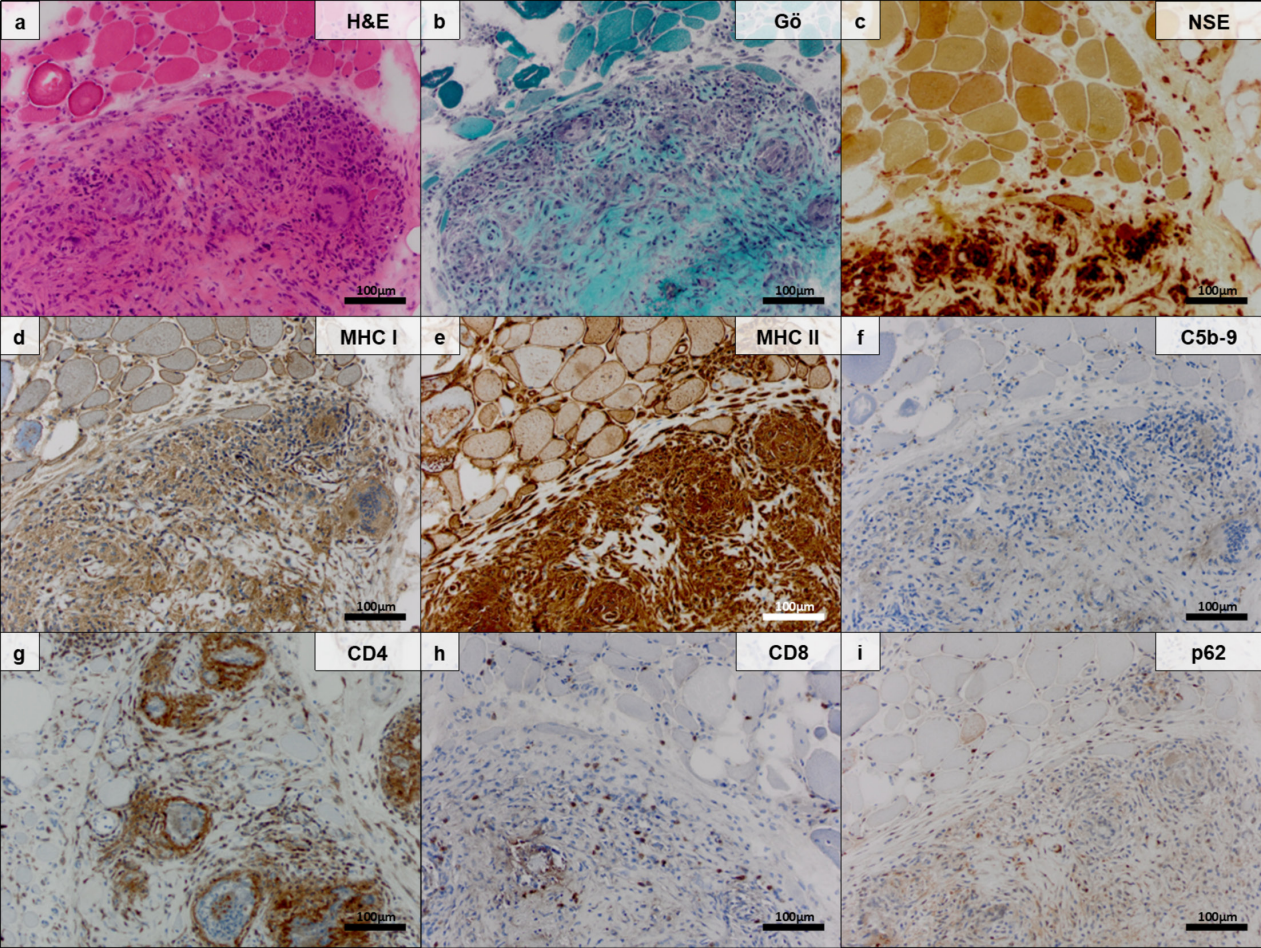
Myopathologic features of a representative sarcoid myopathy specimen with concomitant inclusion body myositis (SaM‐IBM) (original magnification 200×; scale bar 100 μm). Endomysial granuloma containing some giant cells as well as histiocytes/macrophages and T cells dissociating the syncytial arrangement of atrophic myofibres (H&E staining) (a). Endomysial granuloma and some isolated giant cells in severely fibrotic skeletal muscle tissues, characterised by enlarged fibre‐size variation (Gömöri trichrome and nonspecific esterase preparations) (b,c). Macrophages/histiocytes and giant cells as well as the sarcolemma of myofibres, are strongly positive for major histocompatibility complex (MHC) Class I (d) and MHC Cass II (e) (MHC Class I and MHC Class II immunohistochemistry). No major complement deposition on myofibres or capillaries was observed (C5b‐9/MAC immunohistochemistry) (f). The majority of T cells in the vicinity of the granulomas are CD4^+^ and less frequently CD8^+^ as well (CD4 and CD8 immunohistochemistry) (g,h). Presence of coarse autophagic material in myofibres (intravacuolar, subsarcolemmal and perinuclear), as detected by ubiquitin‐binding protein p62 (p62), represents a classic finding in prototypical IBM (p62 immunohistochemistry) (i).

Lymphocytic infiltration in the vicinity of the granulomas with CD4^+^ and CD8^+^ cells, including a predominance of CD4^+^ cells, was observed (Figures 1 and 2) (mean lymphocyte count of 42/HPF). However, an overall predominance of CD4^+^ lymphocytes in the complete muscle biopsy specimen was not evident by conventional morphologic analysis (mean CD4+/CD8+ ratio 1.39, *p* > 0.05) (Table 3).

Immunohistochemistry staining demonstrated no relevant complement deposition (C5‐b9) on myofibres or capillaries of SaM and SaM‐IBM (Figures 1 and 2).

**FIGURE 3:**
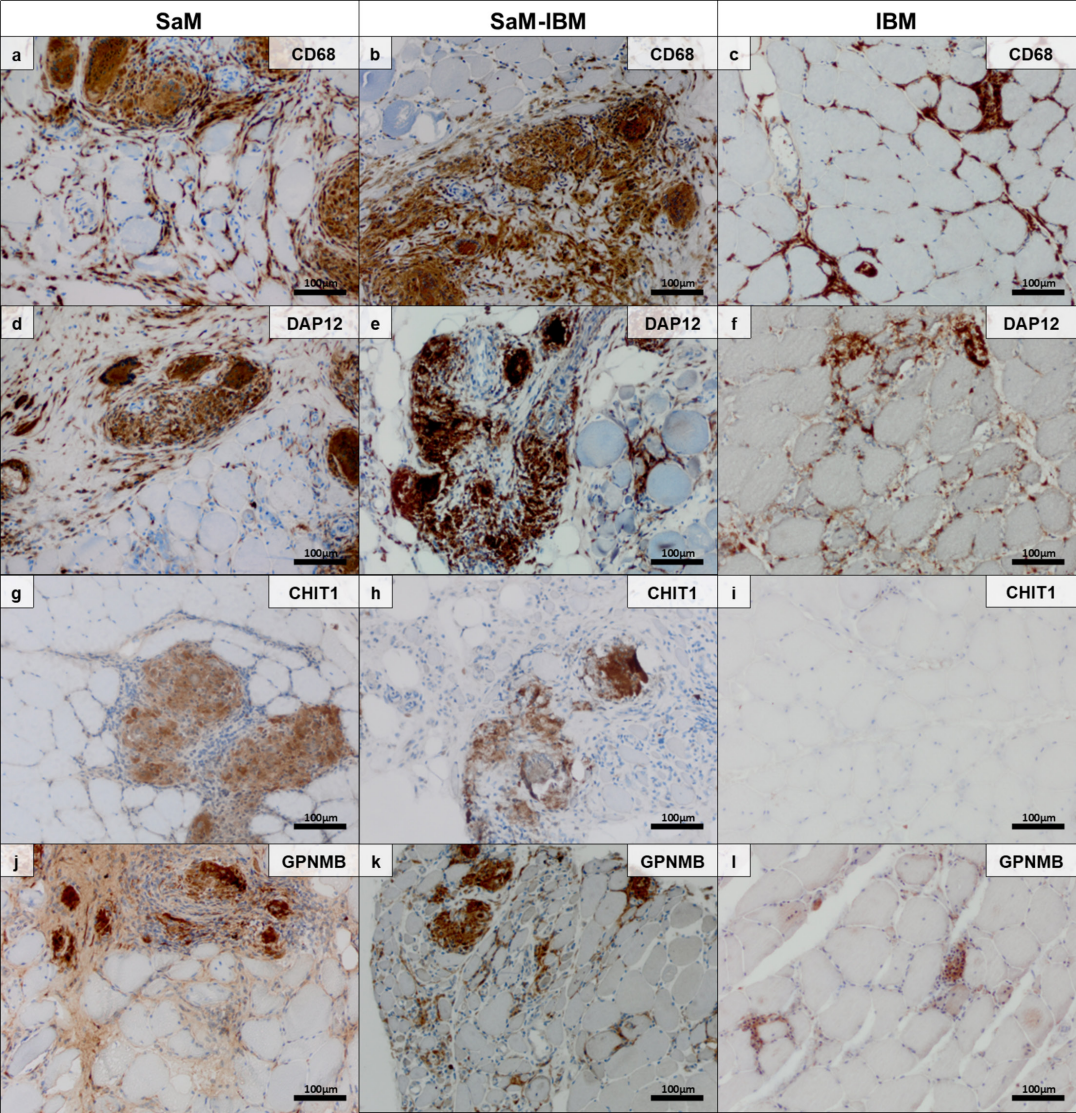
Immunohistochemical characteristics of macrophages and giant cells in granulomatous myositis associated with ‘pure sarcoid myopathy’ (SaM) and SaM with concomitant inclusion body myositis (SaM‐IBM) (original magnification 200×; scale bar 100 μm). Muscle biopsy specimens from patients with ‘pure IBM’ were stained for comparative analysis. CD68^+^ macrophages are detectable in granulomas in giant cells and histiocytes: Macrophages within the endomysium are CD68^+^ in SaM (a) and SaM‐IBM (b) (CD68 immunohistochemistry). In muscle biopsies of ‘pure IBM’, macrophages do not form giant cells and are detectable at high numbers in the endomysium, in myophagocytoses and sometimes within the sarcoplasm, forming small clusters reminiscent of ‘tunnelisation’ (c) (CD68 immunohistochemistry). The essential macrophage fusion competence marker DNAX‐activation protein 12 (DAP12/TYROB) highlights macrophages and giant cells in SaM (d) and SaM‐IBM (e) but also in ‘pure IBM’ (f) (DAP12/TYROB immunohistochemistry). Conversely, chitinase 1 (CHIT1) stains the giant cells in SaM (g) and also in SaM‐IBM (h), whereas macrophages/histiocytes are not stained in both entities and also not in IBM (i) (CHIT1 immunohistochemistry). GPNMB (transmembrane glycoprotein NMB) is a marker of multinucleated giant cells in SaM (j) as well as in SaM‐IBM (k) and is less prominently expressed on macrophages in the vicinity of the granuloma (GPNMB immunohistochemistry). Of note, some scant macrophages were also GPNMB^+^ in IBM muscle tissues (l) (GPNMB immunohistochemistry).

Conventional staining and immunohistochemistry of muscle biopsy specimens from patients with SaM‐IBM showed the same pattern of granulomatous inflammation as seen in ‘pure SaM’ (Figures 2 and S3). However, in contrast to ‘pure SaM’, these biopsies harboured age‐inappropriate amounts of COX‐negative fibres, rimmed vacuoles and p62^+^ aggregates highlighting autoaggressive features, which represent the myopathologic hallmarks of prototypic IBM (Figure 2). The concomitant presence of the clinical IBM phenotype, therefore, does not contradict the proposed classification of two overlapping entities. Also, GPNMB and DAP12/TYROB stained macrophages in IBM, whereas CHIT1 was constantly absent in ‘pure IBM’ muscle biopsy specimens (Figure 3). This observation qualifies CHIT1 as an exclusive giant cell marker that does not differentiate between SaM and SaM‐IBM but between GM as a whole and IBM.

Tables 2 and 3 summarise the morphological features of SaM. Figures 1, 2, 3 illustrate the myopathologic findings in representative biopsy specimens of SaM and SaM‐IBM, including ‘pure IBM’ for comparison.

### 3.3 Ultrastructural Features of SaM and IBM Overlap (SaM‐IBM)

**FIGURE 4:**
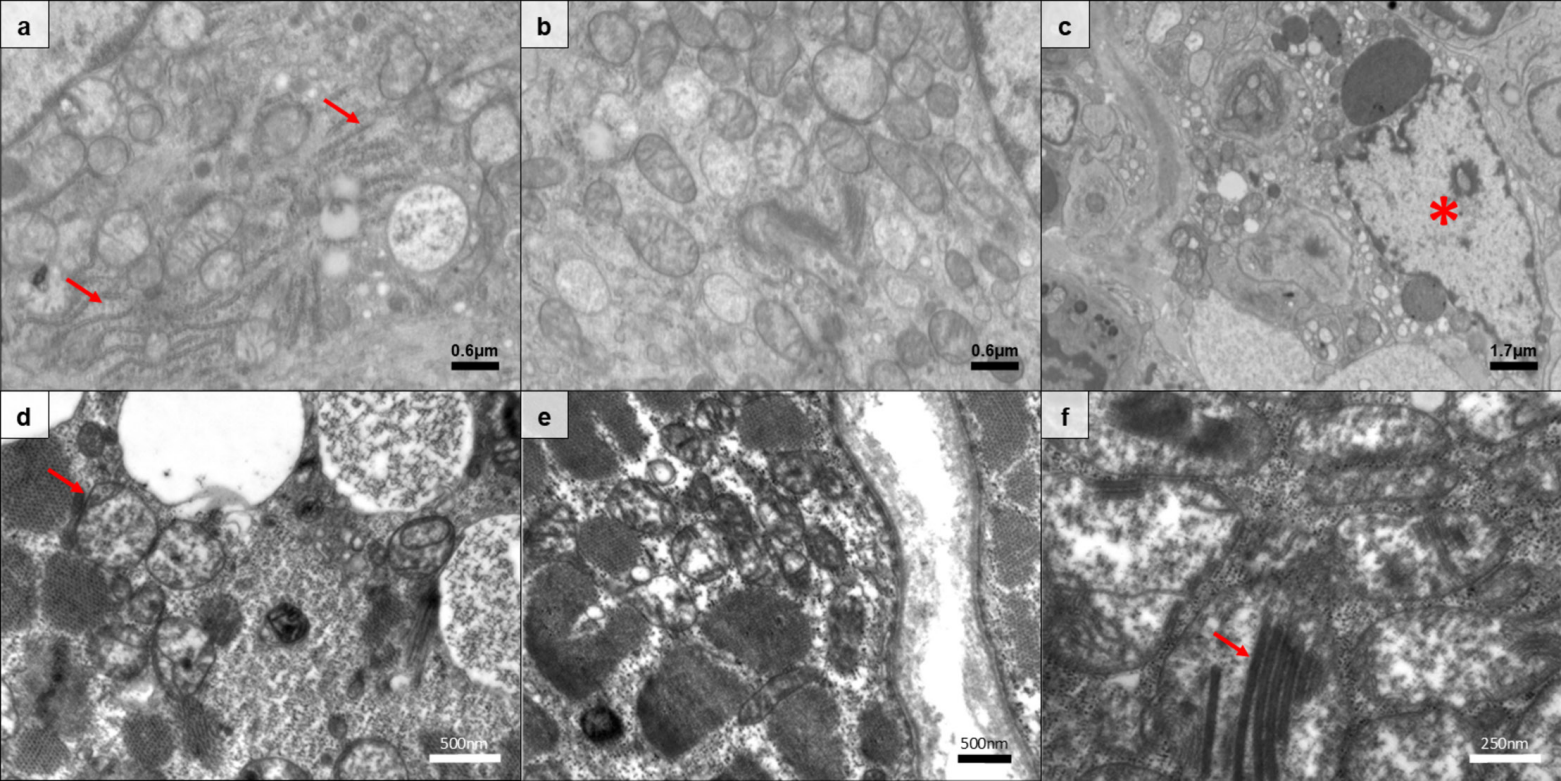
Ultrastructural illustration of subcellular features in ‘pure sarcoid myopathy’ (SaM) and SaM with concomitant inclusion body myositis (SaM‐IBM). Perinuclear (a) and intermyofibrillar (b) accumulation of mitochondria showing regular cristae with some occasional swelling but absence of any pathological cristae formations such as paracrystalline inclusions of circular mitochondrial membranes. Absence of any tubulofilaments in myonuclei (asterisk) or in vacuolar structures that were absent per se (c). Intermyofibrillar accumulation of mitochondria showing circular mitochondrial membranes (arrow) (d), irregular cristae with prominent swelling (e) and paracrystalline inclusions (arrow) (f). *Note:* Presence of some rough endoplasmic reticulum as a broad illustration of cell stress (arrow) (a).

On a subcellular level, ‘pure SaM’ was characterised by focal accumulation of large swollen mitochondria with rare cristae but an absence of irregular cristae (Figure 4). Conversely, SaM‐IBM harboured overt irregular cristae with prominent swelling, circular mitochondrial membranes and paracrystalline inclusions, therefore supporting the diagnosis of IBM harbouring characteristic mitochondrial abnormalities (Figure 4).

### 3.4 Gene Expression in SaM and IBM Overlap (SaM‐IBM) by RT‐qPCR

**FIGURE 5:**
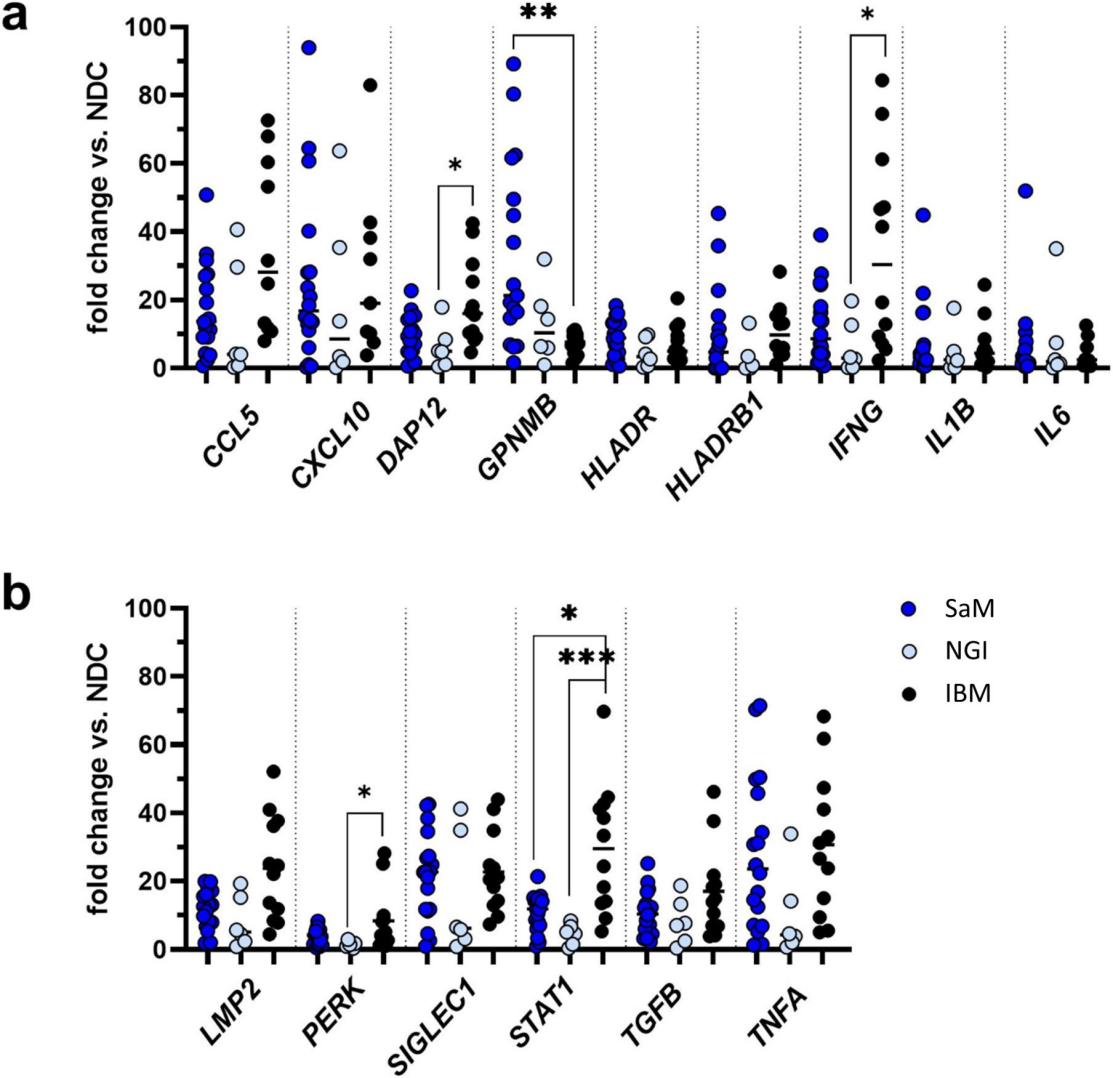
Comparative analysis of gene expression by quantitative real‐time polymerase chain reaction in sarcoid myopathy (SaM), including overlapping inclusion body myositis (SaM‐IBM), muscle biopsies with non‐specific granulomatous inflammation (NGI) (*n* = 6) and ‘pure IBM’ (*n* = 12).

Comparative analysis of SaM and muscle biopsies from patients with non‐specific granulomatous inflammation and IBM demonstrated significant upregulation of *GPNMB* in SaM compared to IBM (Figure 5a). Conversely, gene expression *IFNG*, *STAT1* (signal transducer and activator of transcription 1) and *PERK* (protein kinase R‐like endoplasmic reticulum kinase) were significantly upregulated in ‘pure IBM’, when compared to the non‐specific granulomatous inflammation group. Significance to SaM was only found for *STAT1* (Figure 5). Gene expression profiles of SaM and SaM‐IBM showed no significant differences (Figure S6).

Mitochondrial copy number analysis reveals two distinct patient groups: Both showed reduced mtDNA copy numbers, but to differing degrees. One group exhibited a mild reduction, maintaining mitochondrial expression greater than nuclear expression (mtDNA copy number > 2), whereas the other showed a pronounced reduction with nuclear expression exceeding mitochondrial expression (mtDNA copy number < 2). This was true for both the SaM and non‐specific granulomatous inflammation group (Figure S7a,b).

Importantly, all patients with IBM had strongly reduced mtDNA copy number (mitochondrial expression < nuclear expression), consistent with prior reports of mitochondrial impairment in IBM muscle.

In SaM patients, the copy number also goes along with the expression level of multiple genes, as we identified significant elevation of *CCL5*, *GPNMB*, *IFNG*, *IL1B*, *LMP2*, *PERK*, *SIGLEC1*, *TGFB* and *TNFA* in SaM patients where mitochondrial copy number was pronouncedly reduced (*MTND1* < *B2M*; *n* = 8, including both patients with SaM‐IBM) in comparison to those where mitochondrial copy number was only mildly reduced (*MTND1* > *B2M*; *n* = 10) (Figure S7c,d).

### 3.5 Gene Expression in SaM and IBM Overlap (SaM‐IBM) by RNA Sequencing

**FIGURE 6:**
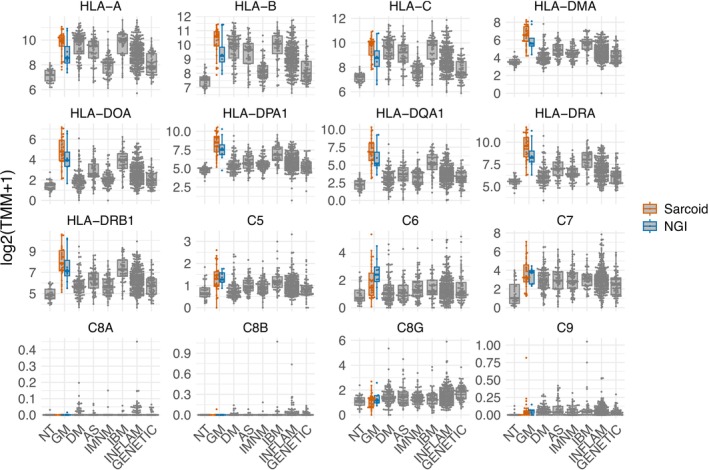
Expression of representative genes in ‘pure sarcoid myopathy’ (SaM), including overlapping inclusion body myositis (SaM‐IBM) and nonspecific granulomatous inflammation (NGI), compared to other myopathies. Each dot represents the gene expression value of a single patient. AS, antisynthetase syndrome; DM, dermatomyositis; GENETIC, genetic myopathies; GM, granulomatous myositis; IBM, inclusion body myositis; INFLAM, inflammatory myopathies; NT, histologically normal muscle biopsies.

**FIGURE 7:**
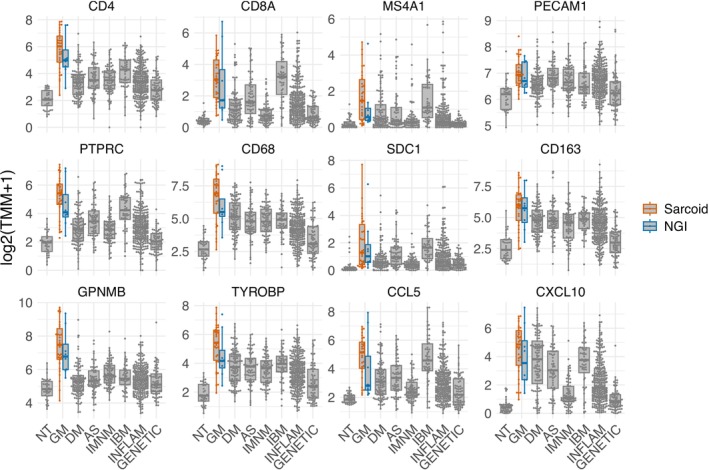
Expression of representative genes in ‘pure sarcoid myopathy’ (SaM), including overlapping inclusion body myositis (SaM‐IBM) and nonspecific granulomatous inflammation (NGI), compared to other myopathies. Each dot represents the gene expression value of a single patient. AS, antisynthetase syndrome; DM, dermatomyositis; GENETIC, genetic myopathies; GM, granulomatous myositis; IBM, inclusion body myositis; INFLAM, inflammatory myopathies; NT, histologically normal muscle biopsies.

**FIGURE 8:**
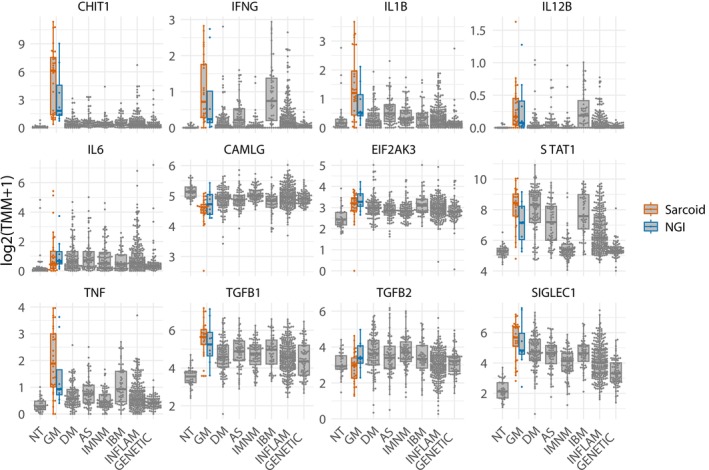
Expression of representative genes in ‘pure sarcoid myopathy’ (SaM) including overlapping inclusion body myositis (SaM‐IBM) and nonspecific granulomatous inflammation (NGI) compared to other myopathies. Each dot represents the gene expression value of a single patient. AS, antisynthetase syndrome; DM, dermatomyositis; GENETIC, genetic myopathies; GM, granulomatous myositis; IBM, inclusion body myositis; INFLAM, inflammatory myopathies; NT, histologically normal muscle biopsies.

Transcriptomic analysis of patients with SaM confirmed markedly elevated expression of both Type 1 and Type 2 human leukocyte antigen (*HLA*) molecules, along with modest increases in the local production of complement pathway components *C5*–*C7* (Figure 6). Macrophage activity markers were elevated more prominently than in any other type of myopathy. T‐cell markers showed increased expression, with *CD4* levels rising relatively more than *CD8*. Similarly, markers for B cells and plasma cells were also elevated (Figure 7). Proinflammatory cytokines including *IFNG*, *IL1β*, *IL6*, *TNFA* and *TGFβ* were consistently upregulated in these patients (Figure 8). Consistent with histologic findings, *CHIT1* was specifically overexpressed in SaM samples but not in ‘pure IBM’ muscle biopsy specimens (Figure 8). Analysis of mitochondrial gene expression revealed a significant decrease in SaM and SaM‐IBM samples with a trend towards SaM‐IBM (Figures S8 and S9).

In line with the previous results of the RT‐qPCR analysis, no relevant differences between patients with SaM and SaM‐IBM were observed.

## 4 Discussion

GM represents the myopathologic hallmark of sarcoidosis but can also rarely develop in other conditions such as IBM [7, 8, 16, 18]. Clinical characterisation of SaM revealed a broad spectrum of phenotypes that frequently include severe motor deficits, specifically if SaM overlaps with IBM [2, 3, 8, 9]. Precise clinical and myopathologic evaluation is needed to identify patients who do not benefit from immunosuppressive therapy introduced for muscle involvement [8]. In this context, our study provides a framework that complements previous clinical studies of SaM [1, 2, 3, 7, 8, 16] and new insights into the immunopathogenesis of GM in sarcoidosis as demonstrated by multidimensional characterisation.

Our results confirm the previously reported clinical presentation of symptomatic SaM [1, 2, 3, 8, 18], which is usually associated with multiorgan disease, including predominant lung involvement, normal CK levels and muscle weakness in most cases. The analysis of muscle biopsy specimens, therefore, represents a crucial part of the diagnostic process to confirm the diagnosis of SaM and exclude other systemic inflammatory conditions, which can present with unremarkable CK levels and muscle weakness, such as vasculitic myopathy [37]. Also, myopathologic confirmation of concomitant IBM in sarcoidosis may inform therapeutic management decisions because these cases show poor treatment response to immunosuppressive therapy [2, 8, 9]. This aspect is of particular importance as the frequency of IBM in SaM seems to be higher than in the general population [2]. For example, SaM with concomitant IBM was present in 2/19 SaM patients from the Berlin cohort in our study, 2/11 SaM patients in the study of Chompoopong et al. [2] and 6/13 patients in the study of Lauletta et al. [8].

The findings from the morphologic analysis expand the spectrum of myopathologic features known to be associated with SaM. The myopathologic features of SaM have not been systematically investigated in larger cohorts to the best of our knowledge, and previous studies essentially describe the presence of perimysial granulomas. Specifically, here we show that SaM is predominantly characterised by endomysial granulomatous inflammation that also commonly involves the perimysial tissue and may extend to the fascia. Additionally, endomysial fibrosis, muscle fibre atrophy, variations of muscle fibre size and capillary thickening can be frequently observed, suggesting a long‐standing and potentially subclinical process of myoinflammation in SaM. Muscle fibre necrosis and internalised myonuclei occur to a lesser extent. Granulomatous structures and muscle fibres in SaM show prominent upregulation of MHC Class I and MHC Class II. This staining pattern complements the previously established diagnostic framework for idiopathic inflammatory myopathies (IIM) [38, 39].

DAP12/TYROB has been characterised as a macrophage fusion regulator in general [40], which also plays a crucial role in SaM as demonstrated by our previous work [10] and immunohistochemical staining in the current study. However, DAP12/TYROB staining is not specific to SaM and can occur in other myopathies as well.

In addition, immunohistochemical staining of GPNMB also highlights giant cells, indicating fusion competence of activated macrophages in SaM. Accordingly, at the gene level, *GPNMB* was significantly upregulated in SaM compared to ‘pure IBM’. Interestingly, *GPNMB* is significantly upregulated in ‘M2‐polarised’ macrophages and has been implicated in inflammatory resolution [41]. Our findings complement our previous work on myoinflammation in SaM, which is driven by Th2‐mediated immune dysregulation [10]. Also, upregulation of GPNMB in giant cells in conjunction with macrophages expressing MHC Class II has been described as a distinct morphological feature that differentiates cardiac sarcoidosis from giant cell myocarditis [24]. However, our investigations rather support a disease‐specific over an organ‐specific pattern of immune dysregulation and inflammation in sarcoidosis, which has been implicated by a recent retrotrans‐genomic analysis [20].

The *CHIT1* gene encodes chitinase 1 (chitotriosidase), which is involved in defence mechanisms of innate immunity and represents a classic biomarker of macrophage activation and maturation. CHIT1 has been recently described as a biomarker of disease activity and severity in pulmonary sarcoidosis [42, 43]. Our results indicate that upregulation of CHIT1 also plays a role in active SaM, suggesting a disease‐specific pattern of immune dysregulation. Furthermore, we have established CHIT1 as a pure giant cell marker in SaM, whereas giant cell markers like DAP12/TYROB and GPNMB are not specific for SaM. In the lung, CHIT1 is expressed in a distinct subgroup of profibrotic macrophages and has been shown to play a central role in pulmonary fibrosis [44]. The CHIT1 positivity of macrophages in SaM may therefore also contribute to endomysial fibrosis, which we observed in the majority of SaM muscle biopsy specimens. Notably, previous in vitro studies showed the efficacy of CHIT1 inhibition in a bleomycin‐induced pulmonary fibrosis model [44, 45]. Taken together, CHIT1 represents a promising therapeutic target in fibrotic sarcoid inflammation.

The observed myopathologic features and immunohistochemical staining patterns provide important diagnostic considerations in cases of suspected IIM. First, the application of CHIT1 staining enables the reliable identification of true giant cells in granulomatous inflammation and may aid the differentiation from so‐called ‘myogenic giant cells’ of non‐histiocytic origin. Second, granulomatous inflammation in SaM may present with a circumscribed pattern, and multilevel sectioning of the muscle biopsy specimen (‘step sections’) can be necessary to identify granulomas. Inflammatory pathology other than granulomatous inflammation may suggest the diagnosis of ‘overlap myositis’ in these cases where granulomas are absent. Notably, hallmark features of specific IIM subtypes, such as perifascicular atrophy or necrosis, diffuse fibre necrosis or p62‐positive fine granular aggregates, were not identified in SaM and SaM‐IBM.

Besides the importance of macrophages in the pathogenesis of SaM, we observed a significant infiltration of T cells, predominantly CD4+ T helper cells. Corresponding to these immunohistochemical findings, *STAT1* was significantly upregulated at the gene level in SaM. These findings are in line with previous research stating the key function of CD4+ cells [46] and dysregulation of Janus kinase–signal transducer and activator of transcription (JAK/STAT) signalling in sarcoidosis [46, 47, 48], therefore providing a pathophysiological rationale for the possible application of JAK inhibition in SaM. However, the efficacy and safety of JAK inhibitors in SaM have not been studied to the best of our knowledge. Interestingly, Damsky et al. [49] reported the resolution of presumably nonspecific focal metabolic avidity in the skeletal muscle of a patient with multiorgan sarcoidosis after the introduction of tofacitinib.

IBM is characterised by a distinct clinical picture that includes severe and progressive motor deficits, particularly pronounced weakness in the deep finger flexors and knee extensors [50]. Notably, and in stark contrast to SaM, there is no extramuscular involvement in IBM. However, as this study demonstrates, IBM can present with different histomorphologic correlates. Two of our SaM patients showed classic clinical (finger flexor weakness), myopathologic and ultrastructural features of IBM and were, therefore, classified as SaM‐IBM. Of note, morphologic evaluation and analysis of gene expression revealed no further differences between ‘pure SaM’ and SaM‐IBM, which further strengthens the argument for shared immune pathologies in SaM‐IBM and sporadic IBM [26]. However, the low number of included SaM‐IBM cases limits this conclusion. Interestingly, accelerated progression to IBM has been recently reported in GM associated with sarcoidosis [8, 9].

Various studies have investigated alterations of mitochondrial copy number levels in human diseases. Mitochondrial diseases—depending on the underlying genetic mutation and pathophysiology—can lead both to a reduction and an increase in mtDNA levels. MtDNA levels in these disorders may vary in multiple organs such as the liver, blood or skeletal muscle [51, 52]. For instance, in mitochondrial encephalomyopathy, lactic acidosis and stroke‐like episodes (MELAS) [53], an elevation of mtDNA copy numbers was described, possibly reflecting a compensatory mechanism. Increasing evidence also suggests that somatic mtDNA mutations could play a role in age‐related neurodegenerative diseases, such as Parkinson's disease [54] and Alzheimer's disease [55] or in certain types of cancer. However, the involvement is heterogeneous and probably depends on the cancer type and the stage [56].

Reduction of mitochondrial copy number is a noteworthy finding, as it can indicate metabolic disturbances. It may indicate reduced mitochondrial gene activity or number, a metabolic shift away from oxidative phosphorylation or a compensatory nuclear response to mitochondrial stress. This would fit with previously reported data on IBM, where reduced mitochondrial copy number likely results from a combination of mtDNA damage and deletions caused by chronic inflammation and oxidative stress, downregulation of mitochondrial biogenesis pathways and a detrimental feedback loop in which mitochondrial dysfunction contributes to progressive muscle fibre degeneration. These findings suggest that mtDNA depletion may have a role in the pathogenesis of the disease [57]. As SaM and IBM show a certain overlap, it is noteworthy that some SaM patients also show a reduction in mtDNA copy number, which may indicate an mtDNA maintenance defect in both diseases [26]. In line with this observation, our transcriptomic analysis revealed a significant decrease of mitochondrial gene expression in SaM and SaM‐IBM. These results complement the previously reported link between mitochondrial dysfunction and *IFNG* upregulation in IIM [58]. Finally, mitochondrial pathology is associated with poor outcome in non‐IBM myositis and may predict progression to IBM [59].

Interestingly, on an ultrastructural level, SaM showed an accumulation of swollen mitochondria, which can be observed in a range of different myopathies and is often considered a non‐specific sign of mitochondrial stress. In SaM‐IBM, however, we observed some of the hallmark signs of mitochondrial disease including irregularities of cristae and widespread paracrystalline inclusions. Of note, these findings were recently described in early and late stages of IBM [25]. The latter may substantiate the speculated disease progression from SaM to IBM. The aforementioned increase of *STAT1* at gene‐expression level in SaM could be explained by elevated interferon type II levels, that is, upregulation of *IFNG* in skeletal muscle with reduced mitochondrial copy numbers. Additionally, *GPNMB* was significantly upregulated in SaM muscle biopsy specimens with reduced mitochondrial copy numbers. GPNMB has been described as an important driver of muscle regeneration and is implicated in the unfolded protein response [60]. Mitochondrial dysfunction in sarcoidosis has been recently studied by different groups, showing mitochondrial tRNA mutations [61] and morphological changes of mitochondria in capillary endothelial cells with disturbed membrane structures in the lung [62]. In line with this, our study also observed ultrastructural mitochondrial changes in SaM patients.

This study has several limitations, including the small sample size and retrospective assessment of patient charts, which was reflected by the incomplete clinical data set (e.g., missing description of muscle weakness severity). Muscle biopsy specimens from patients with infectious myositis, specifically tuberculosis, were not available for this study and would have increased the generalisability of our findings. Finally, the diagnosis of sarcoidosis inherently carries a degree of uncertainty, and other causes may become evident during the follow‐up of a patient.

To conclude, our study outlines the myopathologic and immunologic features of SaM. Histopathologic investigations revealed a stereotypical appearance including endomysial and perimysial granulomatous inflammation frequently extending to the fascia, endomysial fibrosis, muscle fibre atrophy, variations of muscle fibre size and capillary thickening. Notably, mitochondrial dysfunction is evident in both SaM and SaM‐IBM, highlighting the potential role of mitochondria in the disease's pathophysiology. In addition, SaM is characterised by disease‐specific immune dysregulation that involves macrophage function and maturation. Finally, SaM‐IBM represents a noteworthy overlap syndrome that seemingly shares multiple dysregulated immune pathways with ‘pure SaM’.

## Author Contributions

All authors contributed to the study conception, data collection and design. Tissue preparation and analysis were performed by Nikolas Ruffer, Iago Pinal‐Fernandez, Felix Kleefeld, Hans‐Hilmar Goebel, Maria Casal‐Dominguez, Corinna Preusse and Werner Stenzel. The first draft of the manuscript was written by Nikolas Ruffer, Iago Pinal‐Fernandez, Felix Kleefeld, Marie‐Therese Holzer, Werner Stenzel, Corinna Preusse, Udo Schneider and Martin Krusche. All authors commented on previous versions of the manuscript. All authors read and approved the final manuscript.

## Ethics Statement

Ethical approval was granted by the Charité ethics committee (EA2/163/17, AZ 07/09).

## Conflicts of Interest

Werner Stenzel serves as executive editor of *Neuropathology and Applied Neurobiology*. The Editors of *Neuropathology and Applied Neurobiology* are committed to peer‐review integrity and upholding the highest standards of review. As such, this article was peer‐reviewed by independent, anonymous expert referees, and the authors had no role in either the editorial decision or the handling of the paper. The authors declare no conflicts of interest.

## Supporting information


**Figure S1:** Flow chart that displays the included cohorts from the participating institutions [27].

**Figure S3:** Myopathologic features of Patient 2 with sarcoid myopathy and concomitant inclusion body myositis (SaM‐IBM) (original magnification 200×; scale bar 100 μm).

**Figure S4:** Immunofluorescence staining of giant cell markers in ‘pure sarcoid myopathy’ (original magnification 200×; scale bar 100 μm). Double immunofluorescence of chitinase 1 (CHIT1) (Cy3; red channel) (b), DAP12/TYROB (AF488; green channel) (c) and DAPI (nuclei; blue channel) (d) reveal that giant cells co‐stain for macrophage‐fusion‐competence markers DAP12/TYROB and CHIT1 (red arrows), whereas DAP12/TYROB^+^ macrophages at distance from the granuloma residing in the endomysium in a diffuse distribution are negative for CHIT1 (white arrow) (a).

**Figure S5:** Chitinase 1 (CHIT1) is a pure giant cell marker in granulomas of the skeletal muscle (original magnification 200×; scale bar 100 μm). Double immunofluorescence of CHIT1 (Cy3; red channel) (b), CD68 (AF488; green channel) (c) and DAPI (nuclei; blue channel) (d) reveal that giant cells co‐stain CD68 and CHIT1 (red arrows), which represent macrophage markers, whereas macrophages at distance from the granuloma residing in the endomysium in a diffuse distribution are negative for CHIT1 (red arrow) (a).

**Figure S6:** Comparative analysis of gene expression by quantitative real‐time polymerase chain reaction in sarcoid myopathy (SaM) and overlapping inclusion body myositis (SaM‐IBM) revealed no differences between these entities.

**Figure S7:** Comparative analysis of gene expression by quantitative real‐time polymerase chain reaction in sarcoid myopathy including overlapping inclusion body myositis and nonspecific granulomatous inflammation differentiated into patients with increased (green) and reduced (red) mitochondrial copy numbers (a,b). Direct comparison between positive and negative copy numbers in SaM revealed significant differences, as patients with reduced mitochondrial copy numbers demonstrated significant elevated expression levels in multiple genes (c,d).

**Figure S8:** Expression of mitochondrial genes in ‘pure sarcoid myopathy’ (SaM) including overlapping inclusion body myositis (SaM‐IBM) and nonspecific granulomatous inflammation (NGI) compared to other myopathies. Each dot represents the gene expression value of a single patient. AS, antisynthetase syndrome; DM, dermatomyositis; GENETIC, genetic myopathies; GM, granulomatous myositis; IBM, inclusion body myositis; INFLAM, inflammatory myopathies; NT, histologically normal muscle biopsies.

**Figure S9:** Expression of mitochondrial genes in granulomatous myositis separated by inclusion body myositis status compared to other myopathies. Each dot represents the gene expression value of a single patient. AS, antisynthetase syndrome; DM, dermatomyositis; GENETIC, genetic myopathies; GM, granulomatous myositis; IBM, inclusion body myositis; INFLAM, inflammatory myopathies; NT, histologically normal muscle biopsies.

**Table S2:** Summary of primary antibodies used in the study with name, host, clone/clonality, dilution and provider.

**Data S1:** Supporting Information.

## Data Availability

All data relevant to the study are included in the article or uploaded as Supporting Information. No further data are available.
