# Supplementary figures and images for: Myopathology and Immune Profile of Granulomatous Myositis in Sarcoid Myopathy
Source: Neuropathol Appl Neurobiol. 2025 Sep 10;51(5):e70040. doi: 10.1111/nan.70040 (PMC12421948; doi:10.1111/nan.70040)

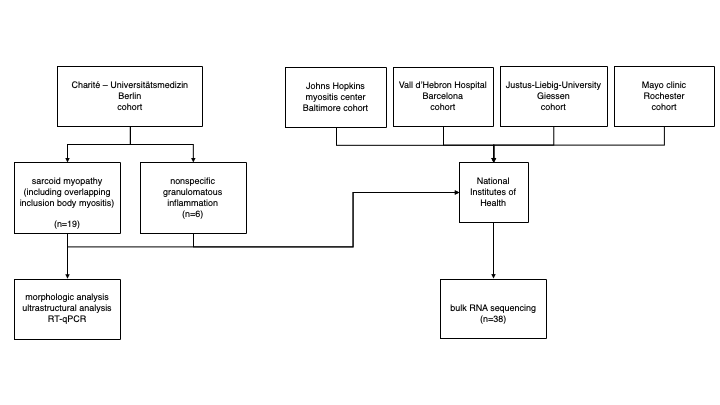

Supplement: Supplementary file 1 — Figure S1: Flow chart that displays the included cohorts from the participating institutions [27]. [file NAN-51-e70040-s009.tiff]

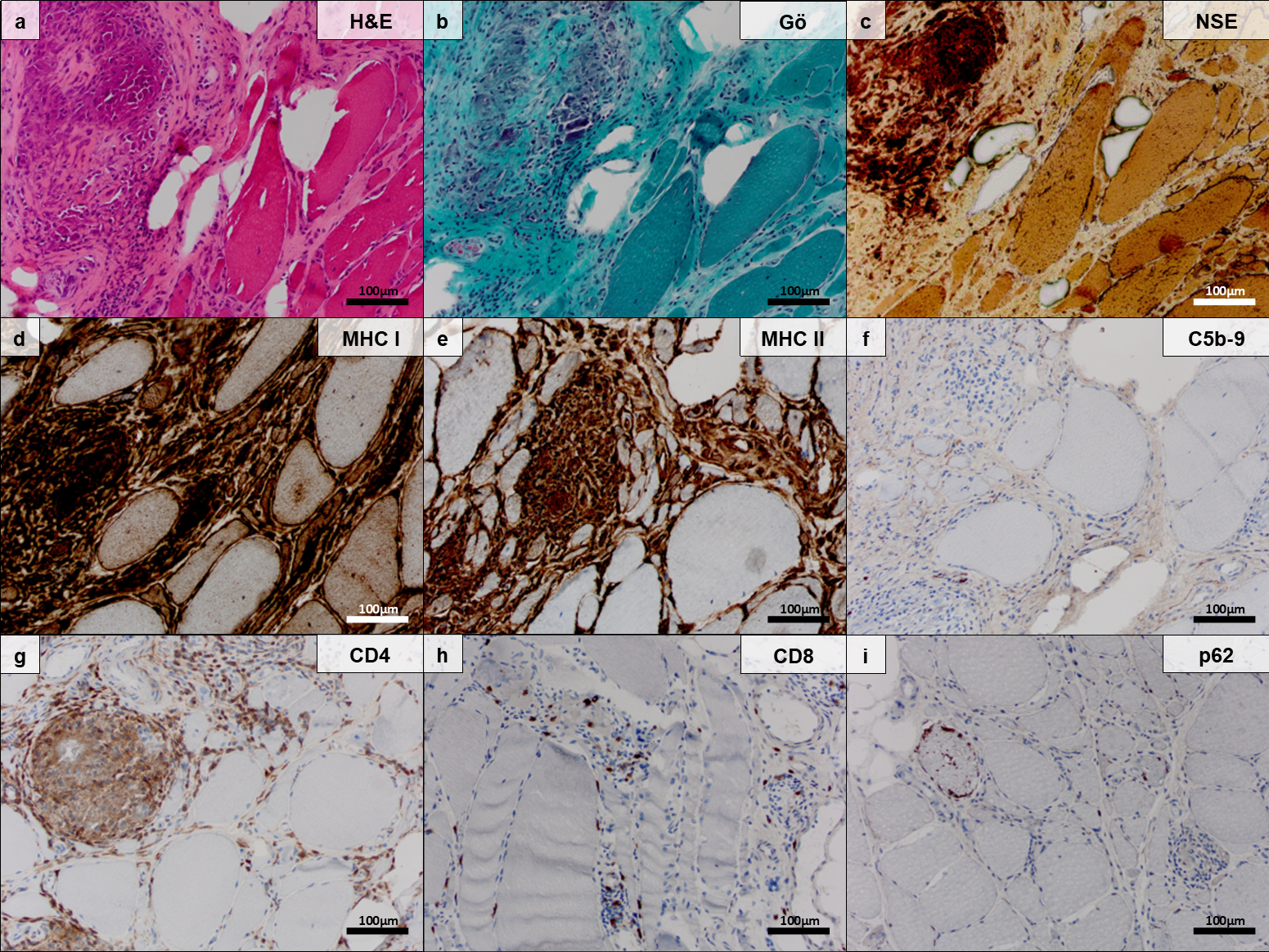

Supplement: Supplementary file 2 — Figure S3: Myopathologic features of Patient 2 with sarcoid myopathy and concomitant inclusion body myositis (SaM‐IBM) (original magnification 200×; scale bar 100 μm). [file NAN-51-e70040-s010.tif]

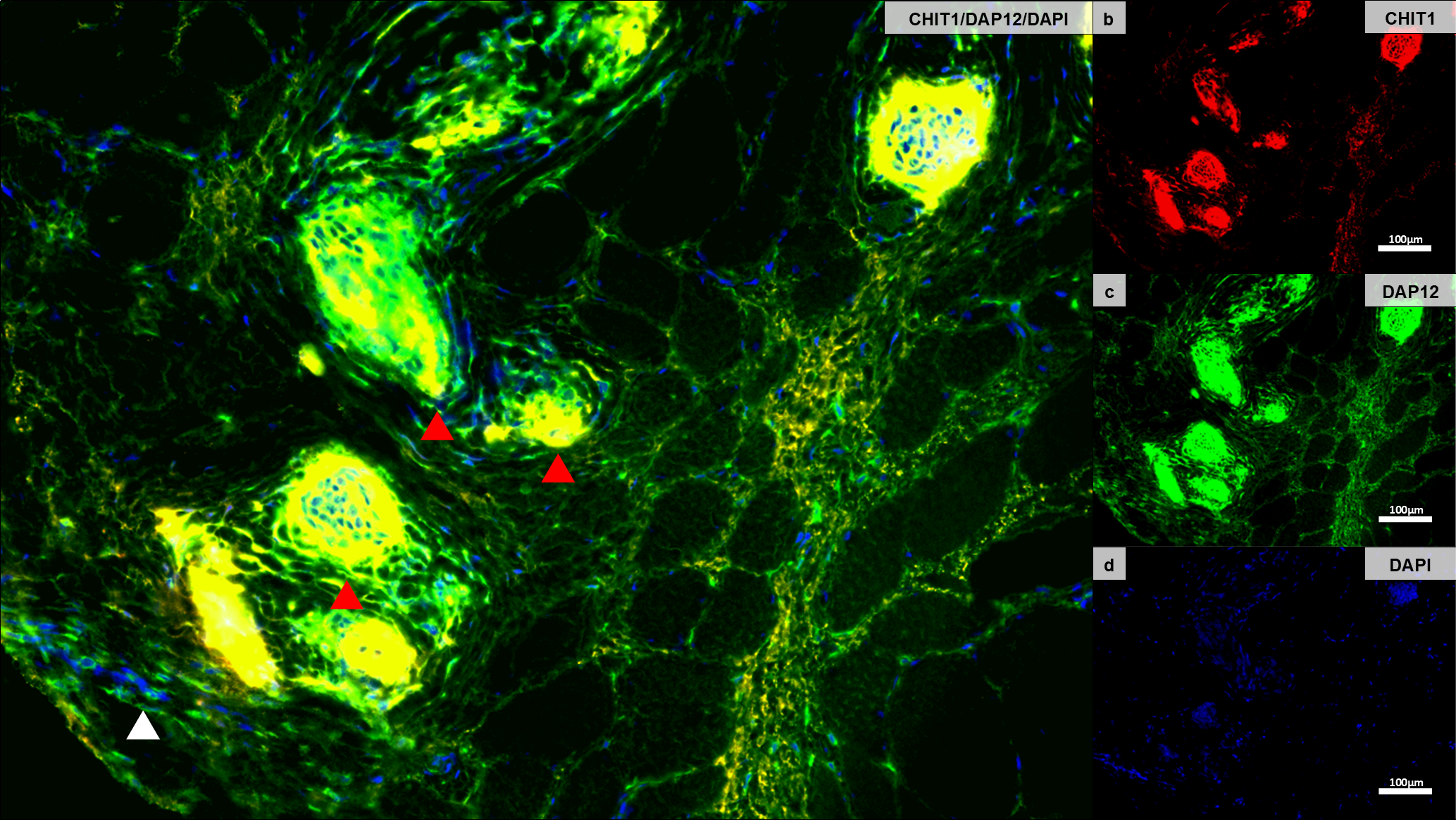

Supplement: Supplementary file 3 — Figure S4: Immunofluorescence staining of giant cell markers in ‘pure sarcoid myopathy’ (original magnification 200×; scale bar 100 μm). Double immunofluorescence of chitinase 1 (CHIT1) (Cy3; red channel) (b), DAP12/TYROB (AF488; green channel) (c) and DAPI (nuclei; blue channel) (d) reveal that giant cells co‐stain for macrophage‐fusion‐competence markers DAP12/TYROB and CHIT1 (red arrows), whereas DAP12/TYROB+ macrophages at distance from the granuloma residing in the endomysium in a diffuse distribution are negative for CHIT1 (white arrow) (a). [file NAN-51-e70040-s008.tif]

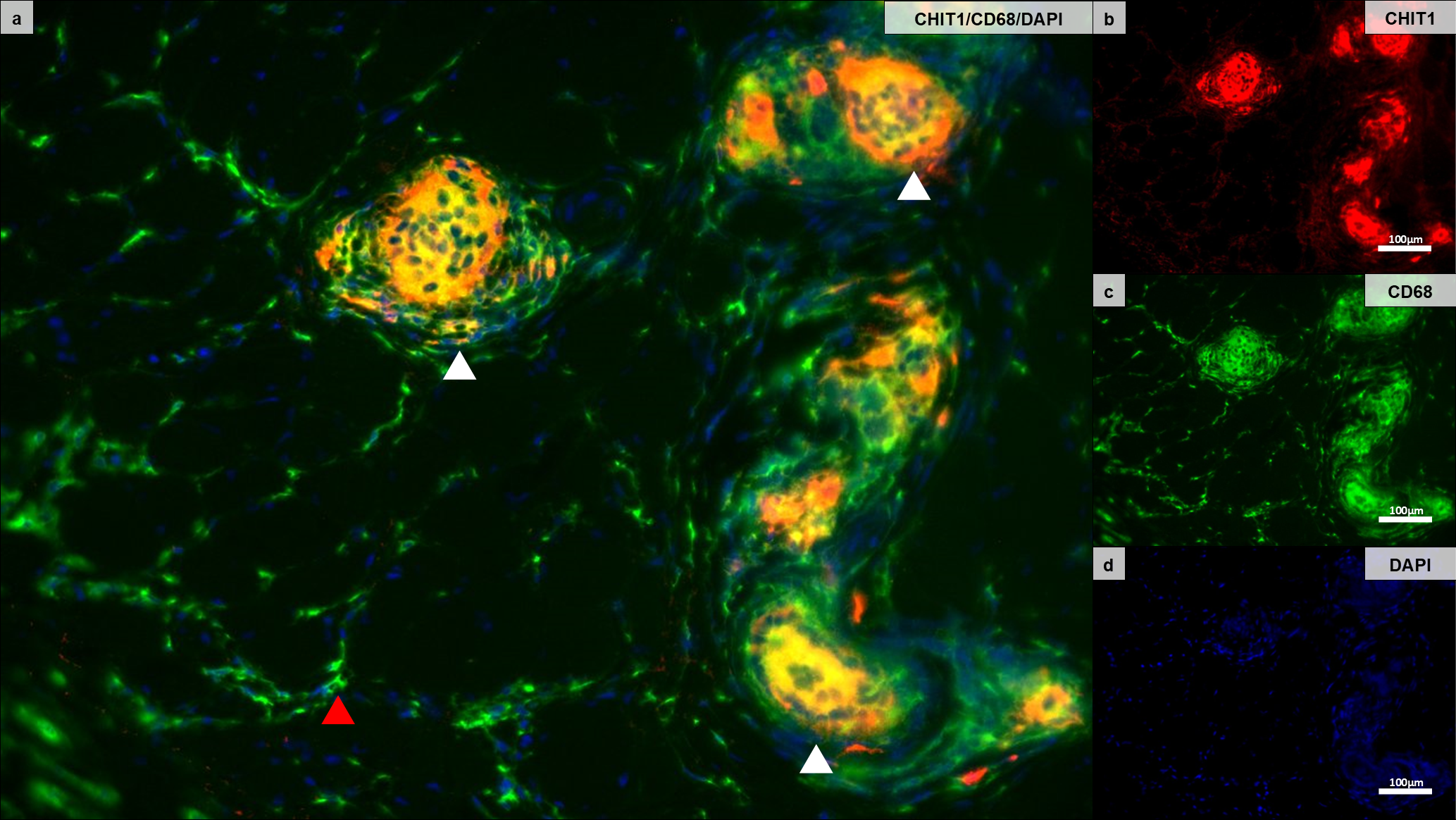

Supplement: Supplementary file 4 — Figure S5: Chitinase 1 (CHIT1) is a pure giant cell marker in granulomas of the skeletal muscle (original magnification 200×; scale bar 100 μm). Double immunofluorescence of CHIT1 (Cy3; red channel) (b), CD68 (AF488; green channel) (c) and DAPI (nuclei; blue channel) (d) reveal that giant cells co‐stain CD68 and CHIT1 (red arrows), which represent macrophage markers, whereas macrophages at distance from the granuloma residing in the endomysium in a diffuse distribution are negative for CHIT1 (red arrow) (a). [file NAN-51-e70040-s002.tif]

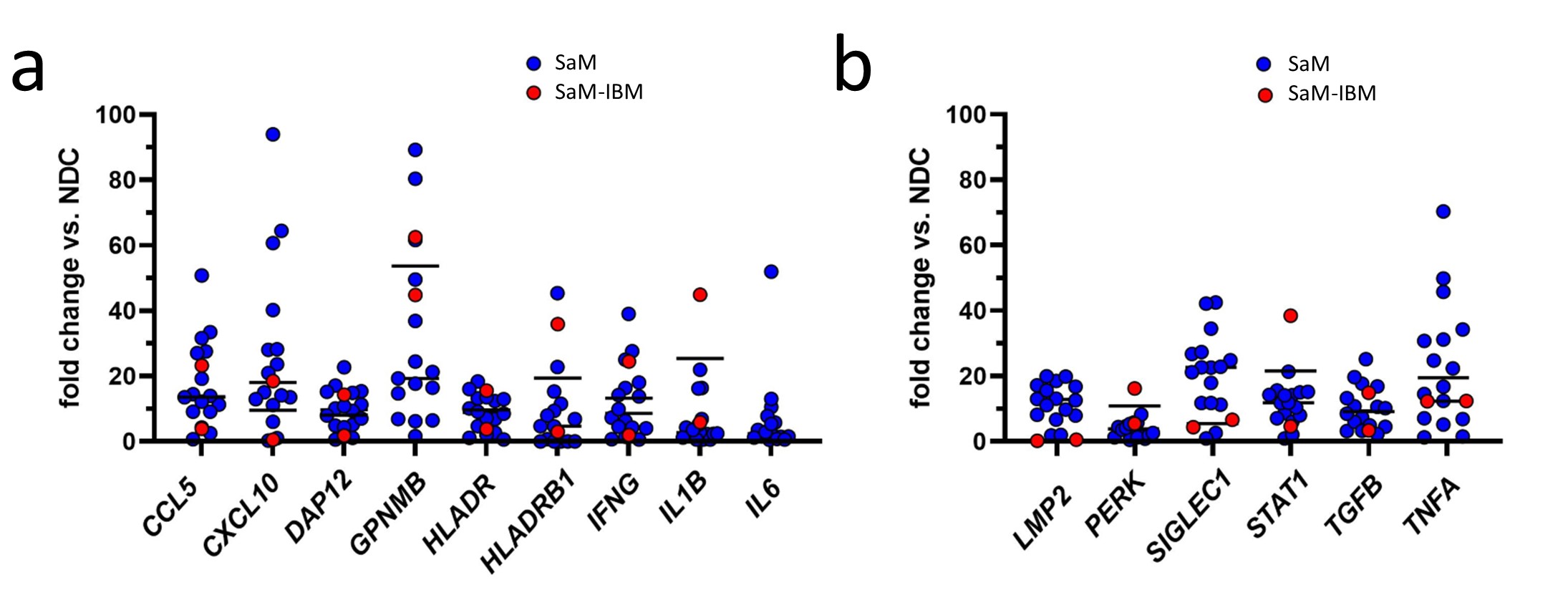

Supplement: Supplementary file 5 — Figure S6: Comparative analysis of gene expression by quantitative real‐time polymerase chain reaction in sarcoid myopathy (SaM) and overlapping inclusion body myositis (SaM‐IBM) revealed no differences between these entities. [file NAN-51-e70040-s003.jpg]

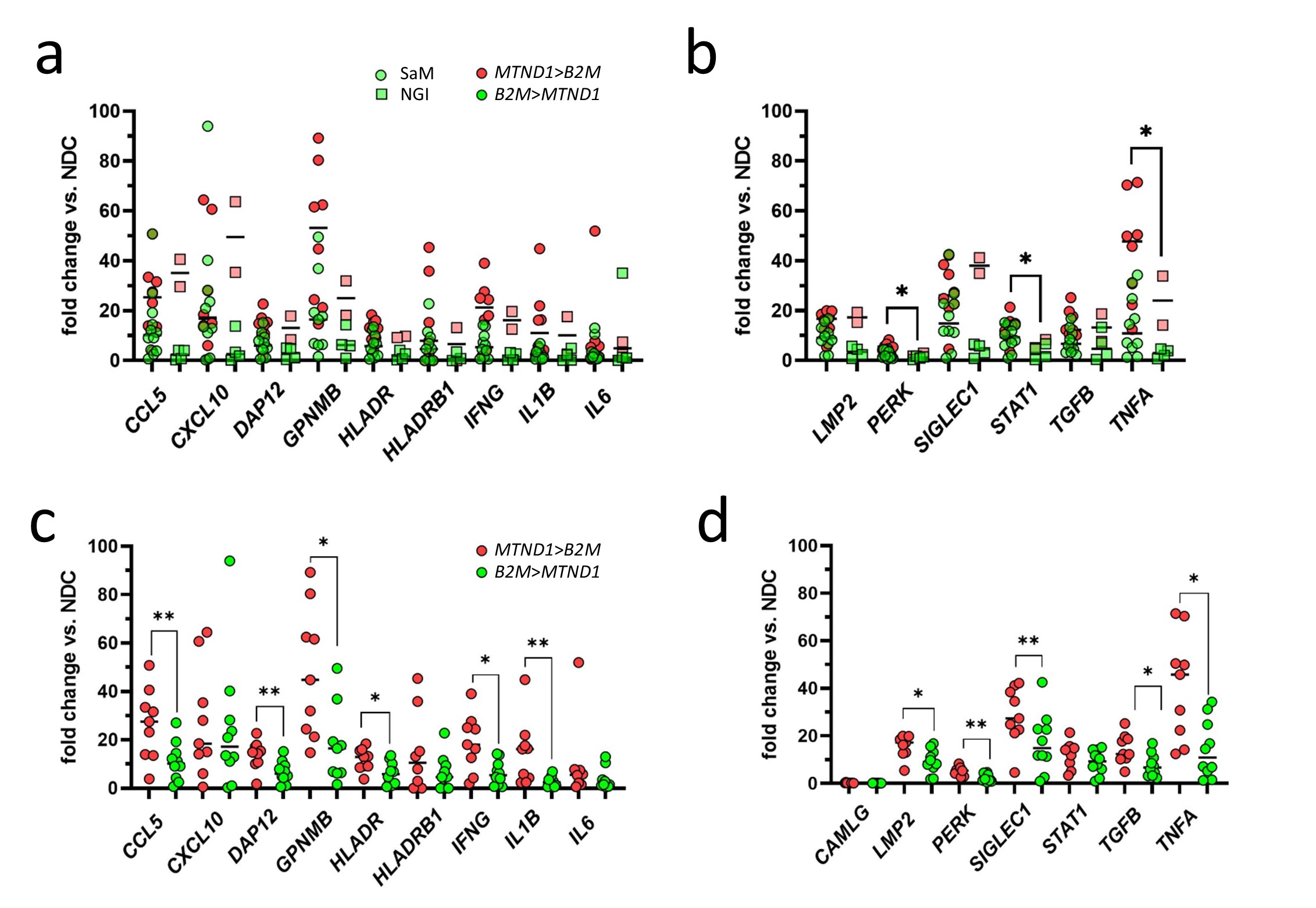

Supplement: Supplementary file 6 — Figure S7: Comparative analysis of gene expression by quantitative real‐time polymerase chain reaction in sarcoid myopathy including overlapping inclusion body myositis and nonspecific granulomatous inflammation differentiated into patients with increased (green) and reduced (red) mitochondrial copy numbers (a,b). Direct comparison between positive and negative copy numbers in SaM revealed significant differences, as patients with reduced mitochondrial copy numbers demonstrated significant elevated expression levels in multiple genes (c,d). [file NAN-51-e70040-s006.jpg]

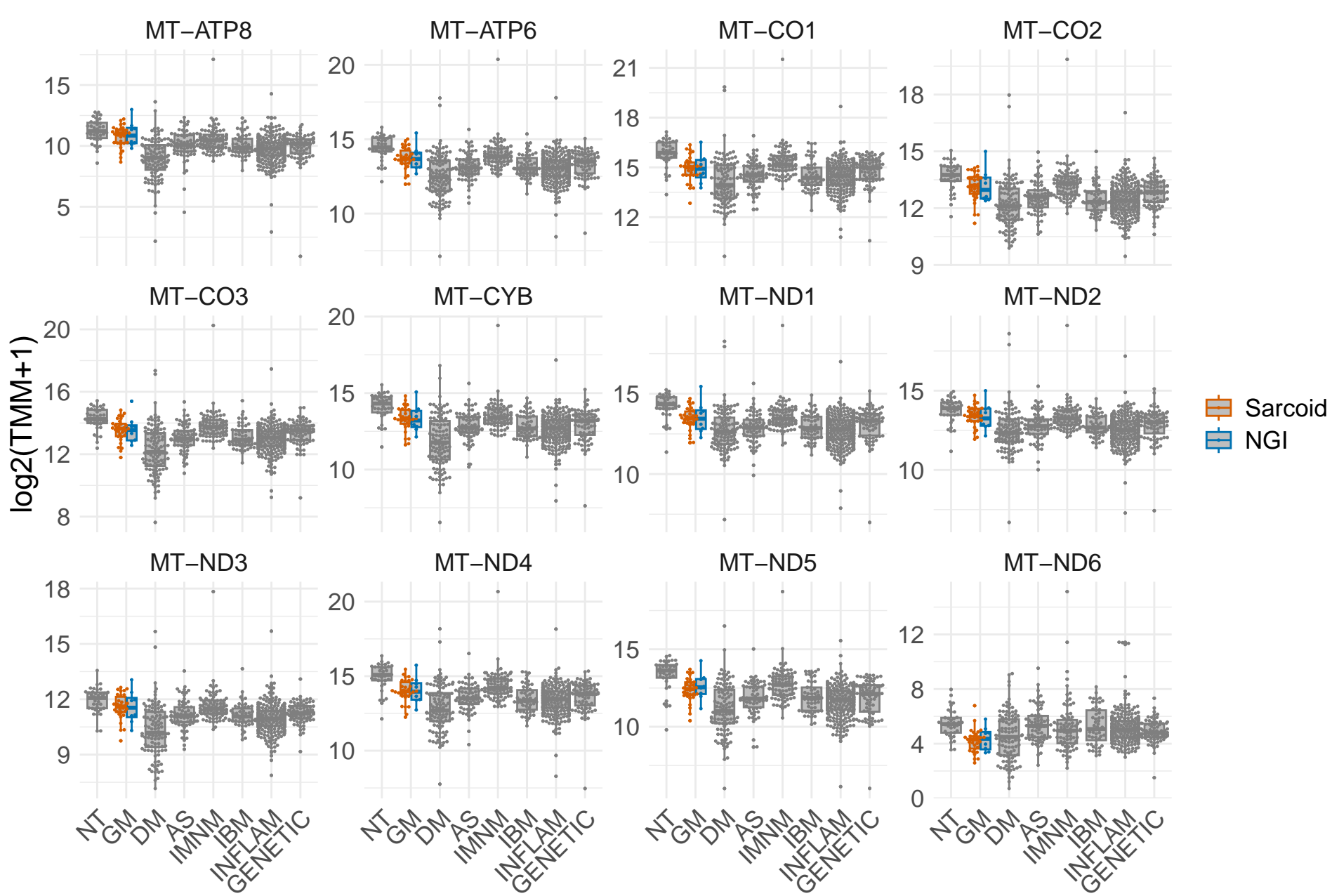

Supplement: Supplementary file 7 — Figure S8: Expression of mitochondrial genes in ‘pure sarcoid myopathy’ (SaM) including overlapping inclusion body myositis (SaM‐IBM) and nonspecific granulomatous inflammation (NGI) compared to other myopathies. Each dot represents the gene expression value of a single patient. AS, antisynthetase syndrome; DM, dermatomyositis; GENETIC, genetic myopathies; GM, granulomatous myositis; IBM, inclusion body myositis; INFLAM, inflammatory myopathies; NT, histologically normal muscle biopsies. [file NAN-51-e70040-s007.pdf]

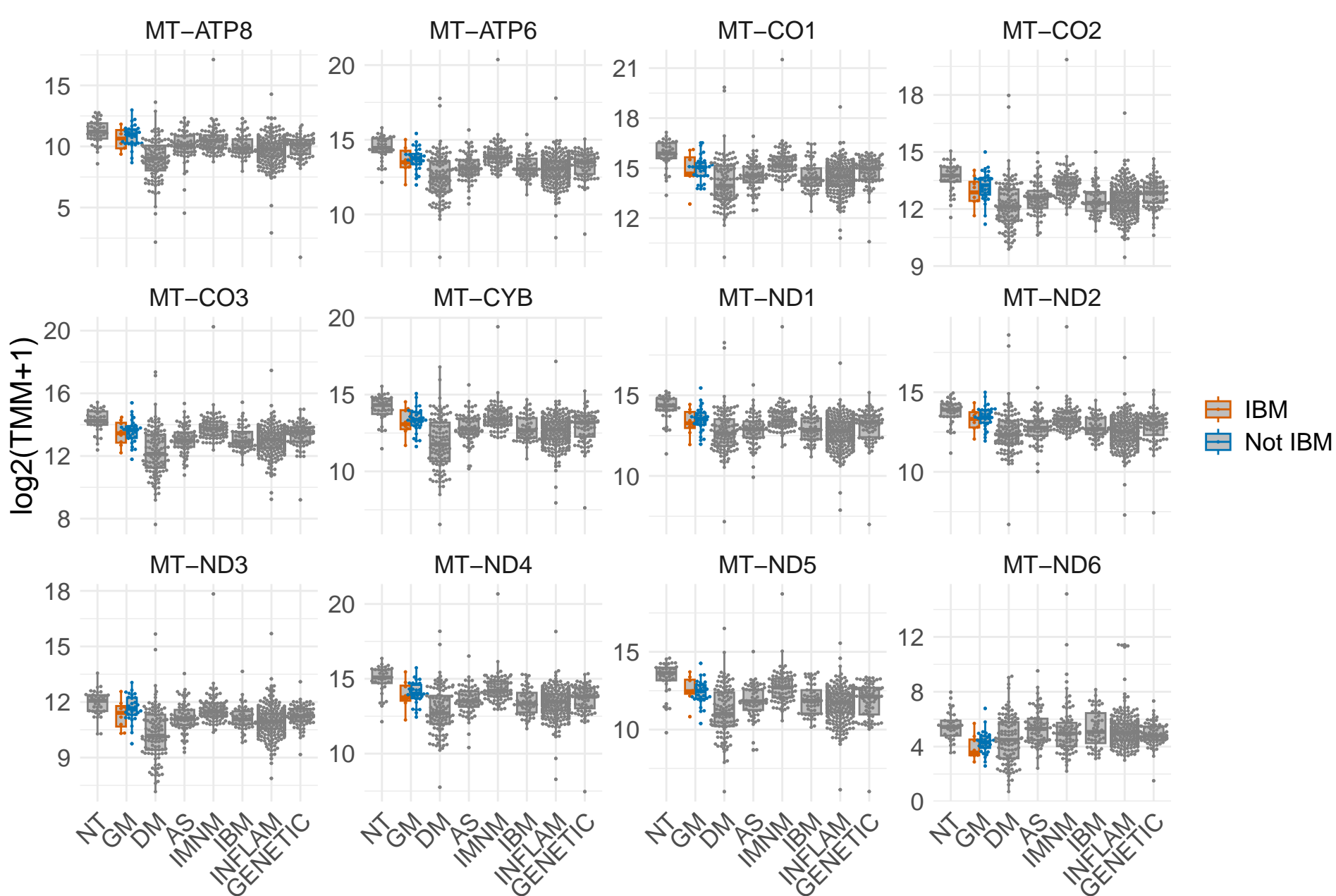

Supplement: Supplementary file 8 — Figure S9: Expression of mitochondrial genes in granulomatous myositis separated by inclusion body myositis status compared to other myopathies. Each dot represents the gene expression value of a single patient. AS, antisynthetase syndrome; DM, dermatomyositis; GENETIC, genetic myopathies; GM, granulomatous myositis; IBM, inclusion body myositis; INFLAM, inflammatory myopathies; NT, histologically normal muscle biopsies. [file NAN-51-e70040-s005.pdf]
