# Supplementary material for: Myopathology and Immune Profile of Granulomatous Myositis in Sarcoid Myopathy
Source: Neuropathol Appl Neurobiol. 2025 Sep 10;51(5):e70040. doi: 10.1111/nan.70040 (PMC12421948; doi:10.1111/nan.70040)
Supplement: Supplementary file 9 — Table S2: Summary of primary antibodies used in the study with name, host, clone/clonality, dilution and provider. [file NAN-51-e70040-s004.docx]

**Supplemental Table S1**. Summary of primary antibodies used in the study with name, host, clone/clonality, dilution and provider.

| Primary antibody | Company | Dilution | Species |
| --- | --- | --- | --- |
| CD4 | Zymed, BRB042 | 1:100 | rabbit |
| CD8 | DAKO, M7050 | 1:100 | mouse |
| CD20 | DAKO, M0755 | 1:200 | mouse |
| CD31 | DAKO, M0823 | 1:100 | mouse |
| CD45 | DAKO, M0701 | 1:400 | mouse |
| CD68 | DAKO, M0718 | 1:100 | mouse |
| CD138 | DAKO, M7228 | 1:30 | mouse |
| CD163 | Quartett, AC-0138 | 1:50 | rabbit |
| C5b-9 | DAKO, M0777 | 1:200 | mouse |
| MHC class I | DAKO, M0736 | 1:1.000 | mouse |
| MHC class II | DAKO, M0775 | 1:100 | mouse |
| GPNMB | Abcam, ab222109 | 1:100 | rabbit |
| DAP-12 | Novus Biologicals, NBP1-85313 | 1:100 | rabbit |
| CHIT1 | Biozol, USB 139700 | 1:100 | rabbit |
| p62 | Abcam, ab 91526 | 1:100 | rabbit |
