# Supplementary material for: Myopathology and Immune Profile of Granulomatous Myositis in Sarcoid Myopathy
Source: Neuropathol Appl Neurobiol. 2025 Sep 10;51(5):e70040. doi: 10.1111/nan.70040 (PMC12421948; doi:10.1111/nan.70040)
Supplement: Supplementary file 10 — Data S1: Supporting Information. [file NAN-51-e70040-s001.docx]

**SUPPLEMENTAL MATERIAL**

**Supplementary Fig. S1**. Flow chart that displays the included cohorts from the participating institutions [27].

**
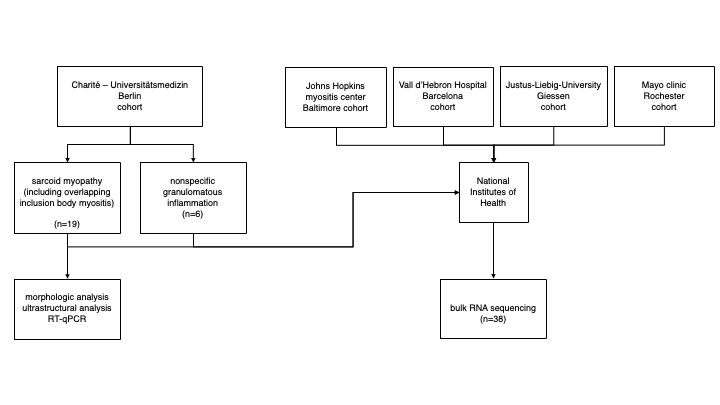
**

**Supplementary Table S2**. Summary of primary antibodies used in the study with name, host, clone/clonality, dilution and provider.

| Primary antibody | Company | Dilution | Species |
| --- | --- | --- | --- |
| CD4 | Zymed, BRB042 | 1:100 | rabbit |
| CD8 | DAKO, M7050 | 1:100 | mouse |
| CD20 | DAKO, M0755 | 1:200 | mouse |
| CD31 | DAKO, M0823 | 1:100 | mouse |
| CD45 | DAKO, M0701 | 1:400 | mouse |
| CD68 | DAKO, M0718 | 1:100 | mouse |
| CD138 | DAKO, M7228 | 1:30 | mouse |
| CD163 | Quartett, AC-0138 | 1:50 | rabbit |
| C5b-9 | DAKO, M0777 | 1:200 | mouse |
| MHC class I | DAKO, M0736 | 1:1.000 | mouse |
| MHC class II | DAKO, M0775 | 1:100 | mouse |
| GPNMB | Abcam, ab222109 | 1:100 | rabbit |
| DAP-12 | Novus Biologicals, NBP1-85313 | 1:100 | rabbit |
| CHIT1 | Biozol, USB 139700 | 1:100 | rabbit |
| p62 | Abcam, ab 91526 | 1:100 | rabbit |


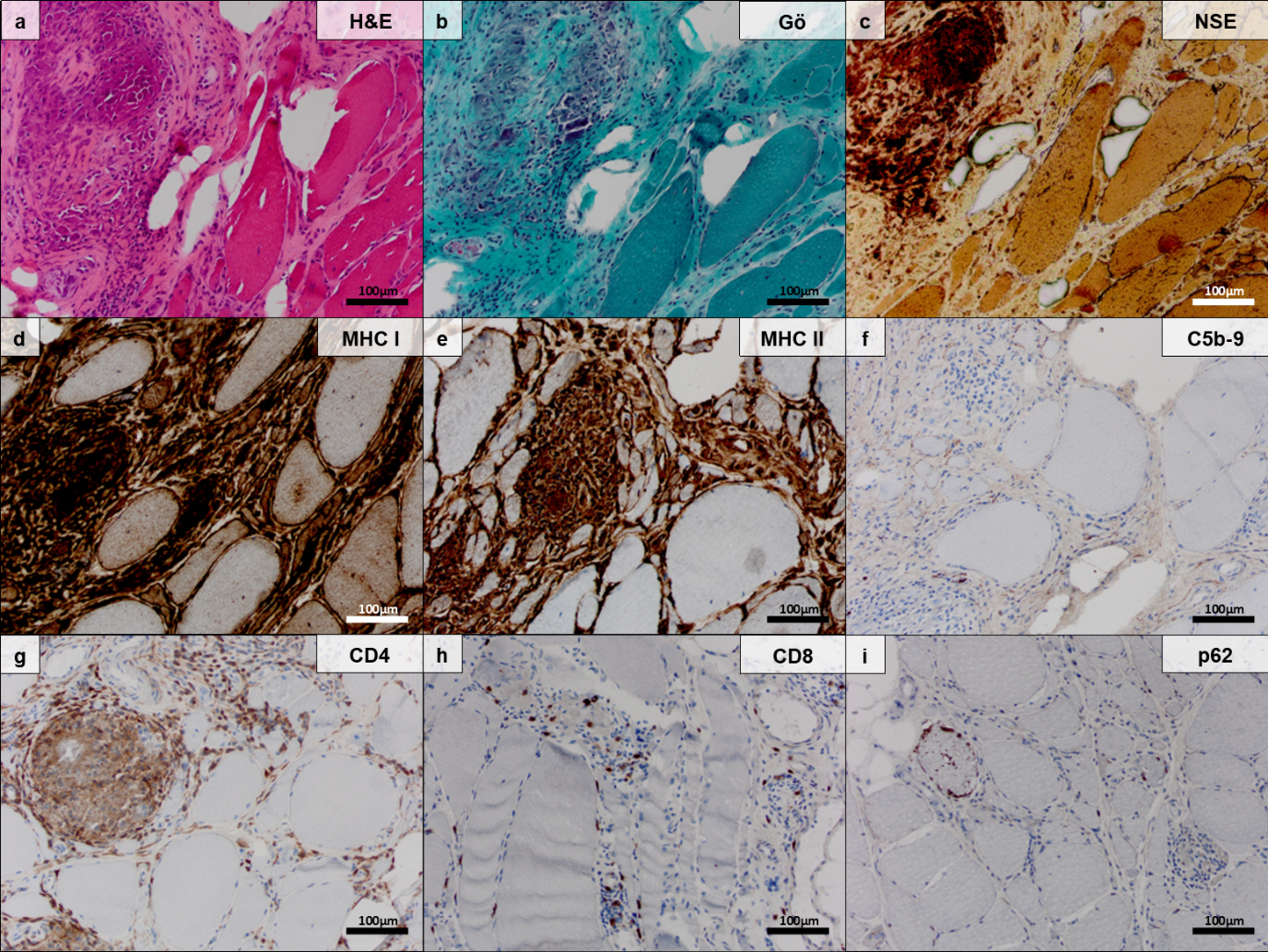


**Supplementary Fig. S3**. Myopathologic features of patient 2 with sarcoid myopathy and concomitant inclusion body myositis (SaM-IBM) (original magnification x200; scale bar 100 µm).


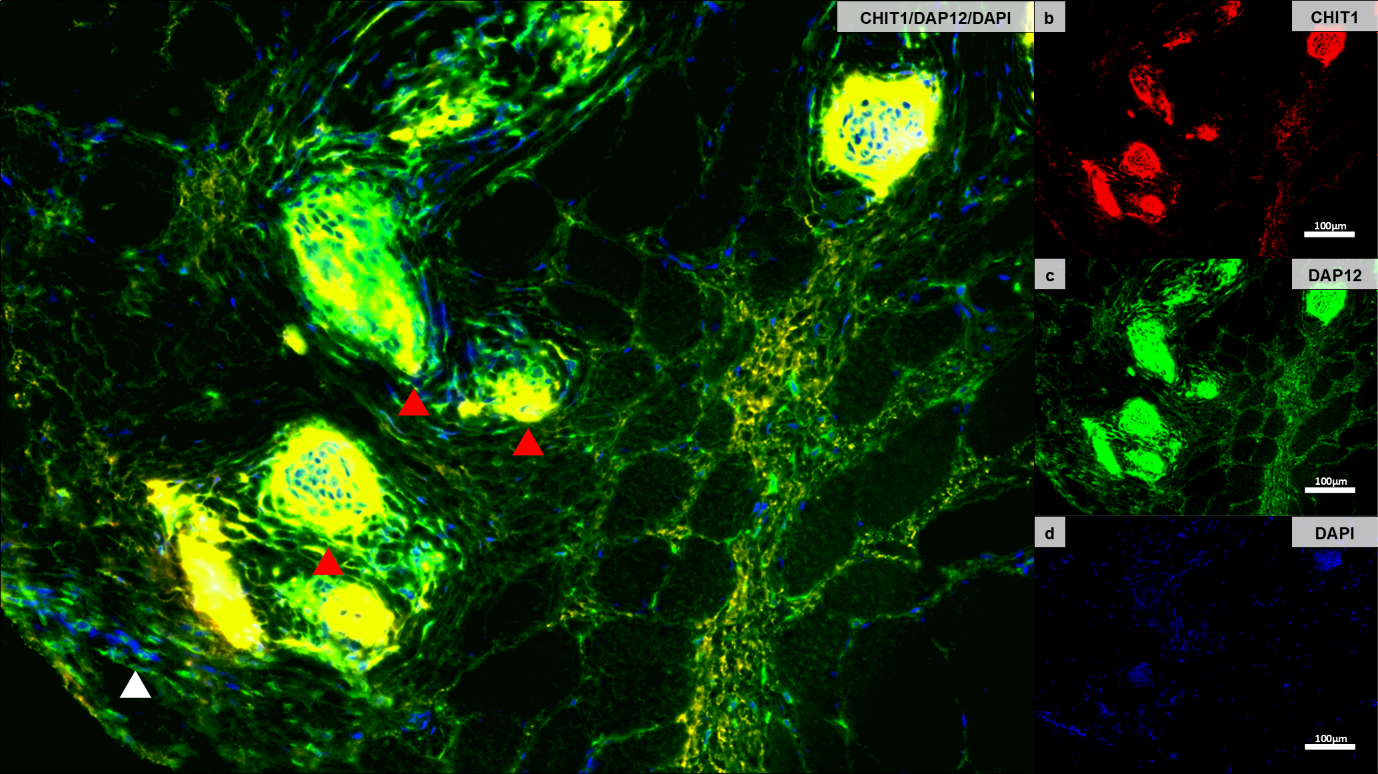


**Supplementary Fig. S4**. Immunofluorescence staining of giant cell markers in ‘pure sarcoid myopathy’ (original magnification x200; scale bar 100 µm). Double immunofluorescence of Chitinase 1 (CHIT1) (Cy3; red channel) (**b**), DAP12/TYROB (AF488; green channel) (**c**) and DAPI (nuclei; blue channel) (**d**) reveal that giant cells co-stain for macrophage-fusion-competence markers DAP12/TYROB and CHIT1 (red arrows), while DAP12/TYROB^+^ macrophages at distance from the granuloma residing in the endomysium in a diffuse distribution are negative for CHIT1 (white arrow) (**a**).


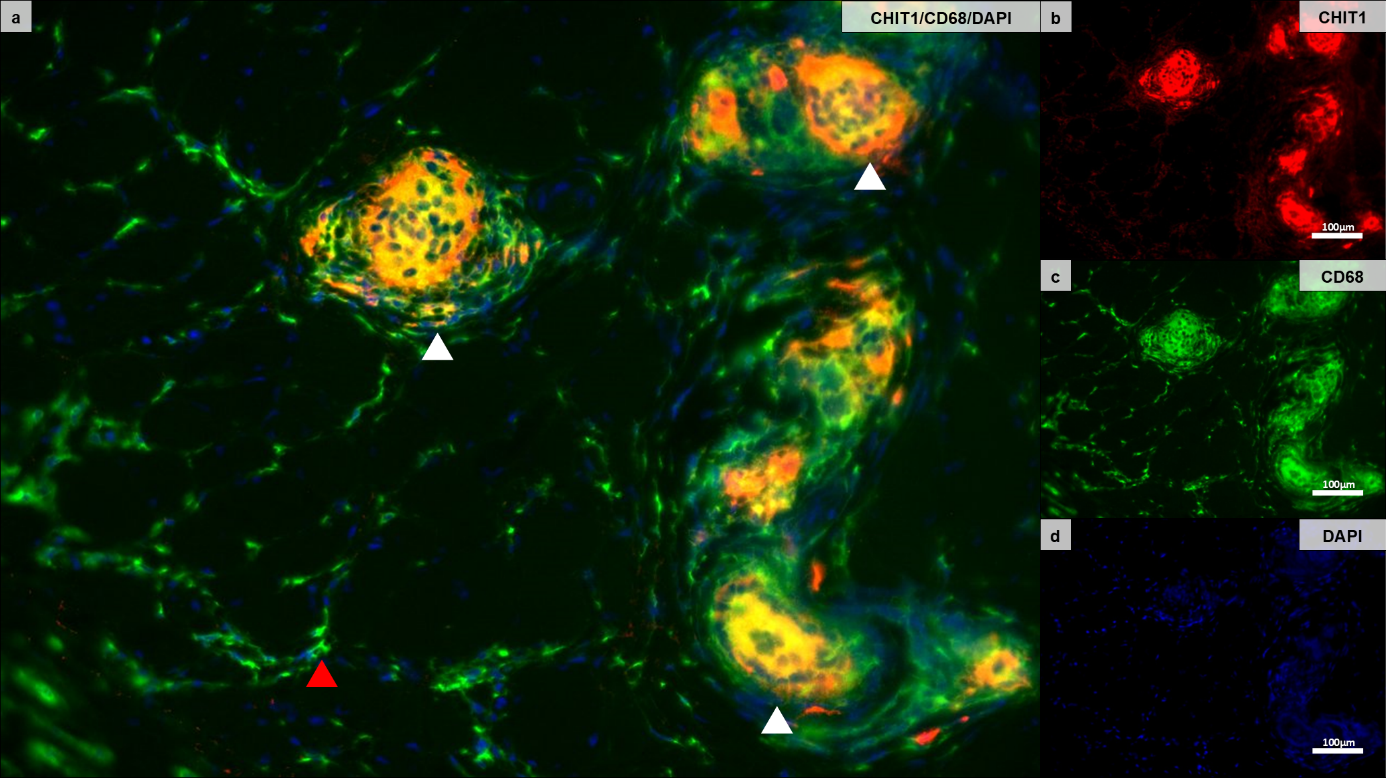


**Supplementary Fig. S5**. Chitinase 1 (CHIT1) is a pure giant cell marker in granulomas of the skeletal muscle (original magnification x200; scale bar 100 µm). Double immunofluorescence of CHIT1 (Cy3; red channel) (**b**), CD68 (AF488; green channel) (**c**) and DAPI (nuclei; blue channel) (**d**) reveal that giant cells co-stain CD68 and CHIT1 (red arrows), which represent macrophage markers, while macrophages at distance from the granuloma residing in the endomysium in a diffuse distribution are negative for CHIT1 (red arrow) (**a**).


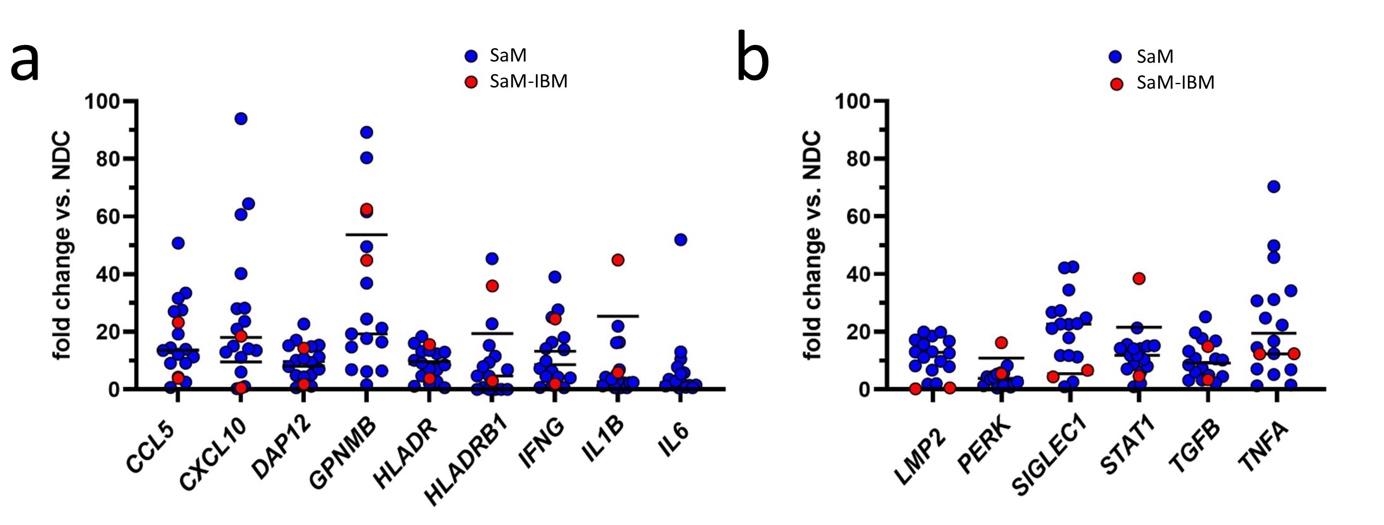


**Supplementary Fig. S6**. Comparative analysis of gene expression by quantitative real-time polymerase chain reaction in sarcoid myopathy (SaM) and overlapping inclusion body myositis (SaM-IBM) revealed no differences between these entities.

**
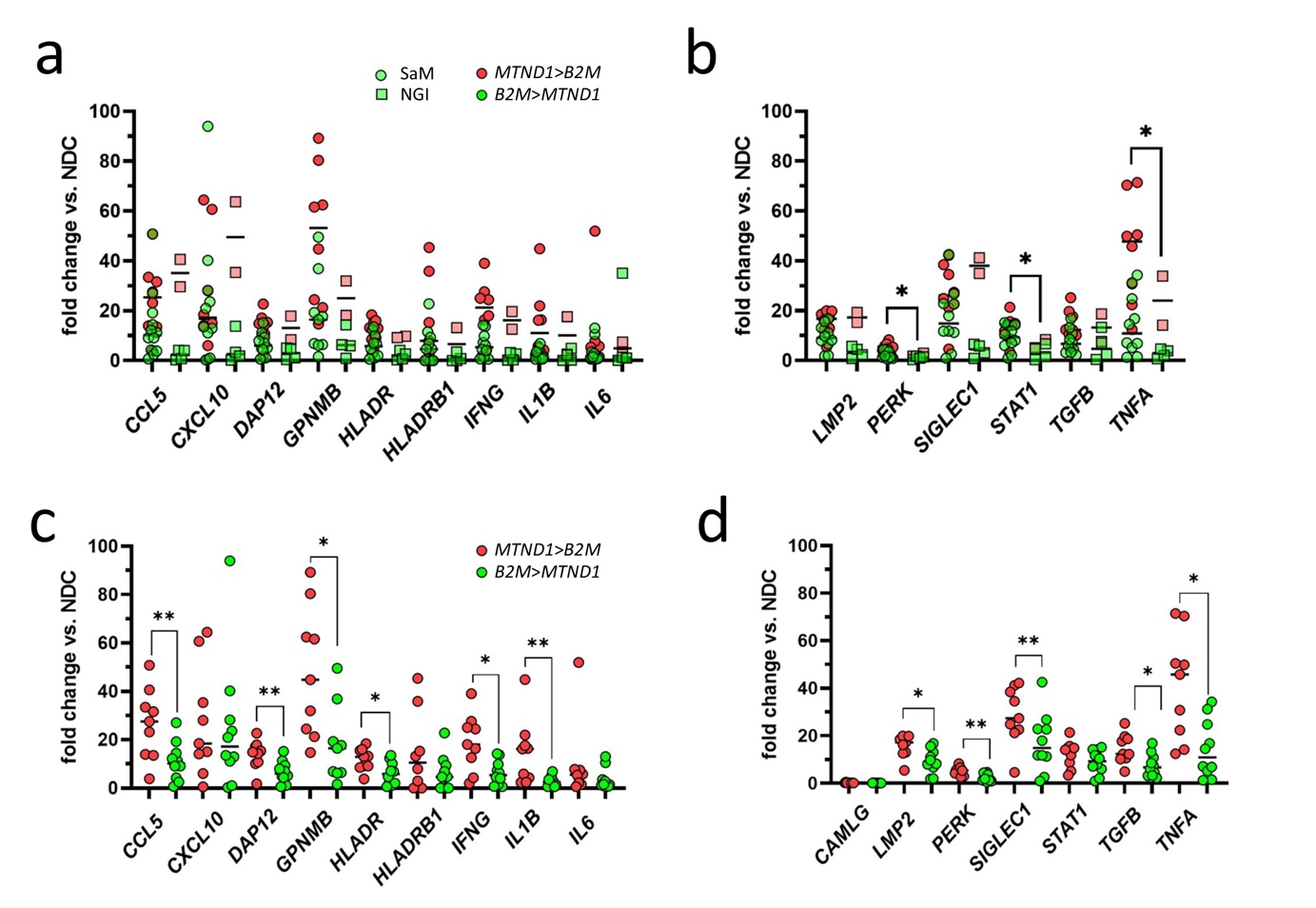
**

**Supplementary Fig. S7**. Comparative analysis of gene expression by quantitative real-time polymerase chain reaction in sarcoid myopathy including overlapping inclusion body myositis and nonspecific granulomatous inflammation differentiated into patients with increased (green) and reduced (red) mitochondrial copy numbers (**a**, **b**). Direct comparison between positive and negative copy numbers in SaM revealed significant differences, as patients with reduced mitochondrial copy numbers demonstrated significant elevated expression levels in multiple genes (**c**, **d**).

**Supplementary Fig. S8**. Expression of mitochondrial genes in ‘pure sarcoid myopathy’ (SaM) including overlapping inclusion body myositis (SaM-IBM) and nonspecific granulomatous inflammation (NGI) compared to other myopathies. Each dot represents the gene expression value of a single patient. NT: histologically normal muscle biopsies; GM: granulomatous myositis; DM: dermatomyositis; AS: antisynthetase syndrome; IBM: inclusion body myositis; INFLAM: inflammatory myopathies; GENETIC: genetic myopathies.

**Supplementary Fig. S9**. Expression of mitochondrial genes in granulomatous myositis separated by inclusion body myositis status compared to other myopathies. Each dot represents the gene expression value of a single patient. NT: histologically normal muscle biopsies; GM: granulomatous myositis; DM: dermatomyositis; AS: antisynthetase syndrome; IBM: inclusion body myositis; INFLAM: inflammatory myopathies; GENETIC: genetic myopathies.
